# Cognitive processes of ingroup favoritism across 20 countries: An eye-tracking investigation of culture, behavior, and cognition

**DOI:** 10.1073/pnas.2417456122

**Published:** 2025-08-05

**Authors:** Rima-Maria Rahal, Frederik Schulze Spüntrup

**Affiliations:** ^a^Behavioral Law and Economics, Max Planck Institute for Research on Collective Goods, Bonn 53133, Germany; ^b^Institute for Cognition and Behavior, Vienna University of Economics and Business, Wien 1020, Austria; ^c^Institute for Globally Distributed Open Research and Education (IGDORE), Göteborg 413 90, Sweden

**Keywords:** discrimination, prosocial behavior, in-group favoritism, social cognition, cross-cultural

## Abstract

Whether and to what degree culture modifies cognition has been an area of research often limited by possibilities to gather relevant data across societies. In this project, we leverage webcam-based eye-tracking to study cultural variations of cognitive processes underlying in-group favoritism. Participants (n = 1850, k = 20) are assigned to an in-group based on a color perception task, complete a group reinforcement stage, and then make decisions to allocate points between themselves and random matched players in a repeated decomposed dictator game, facing either an in- or an out-group member. During this task, we recorded eye-gaze with participants’ webcams. Results show substantial variation in behavioral in-group favoritism across cultures, linking discrimination based on group membership to higher societal uncertainty. Individual preferences for prosociality conditioned differences in visual search effort, but the directionality of the effect differs between countries. Individual-level predictors explained participants remaining colorblind toward others’ group membership, where society-level predictors did not. Overall, we demonstrate the importance of taking culture into account when assessing cognitive processes underlying behavior. Implications for globally oriented policymaking to reduce discrimination are discussed.

## Significance

This study examines how cultural contexts shape the way that decision-makers reason about decisions to discriminate against out-groups, capturing visual attention to decision-relevant cues via webcam-based eye-tracking from 1,850 participants across 20 countries. Results show that while individual preferences for prosociality influence visual attention, the direction of these effects varies across cultures. The study highlights that societal uncertainty correlates with increased group-based discrimination, offering insights into the mechanisms behind cross-cultural differences in social cognition. These findings advance our understanding of discrimination in favor of the in-group and emphasize the importance of cultural considerations in developing policies aimed at reducing discrimination globally, making this research significant for both the academic community and policymakers.

In times of global polycrisis, cooperation across borders is central to human well-being and survival. However, real-world behavior often fails to reach cooperative states—particularly when the activation of in-group bias kicks in (1)—with tragic consequences. In the scope of the COVID-19 pandemic, inequitable distribution of personal protective equipment, respirators, and vaccines aggravated the spread and death-toll associated with the disease (2, 3): Western nations poured resources into fighting COVID-19 to aid their own populations, securing the high proportions of global supplies to fulfill their own demands and only thereafter distributing vaccines to developing countries. The climate crisis finds individuals shirking responsibility for taking action today to benefit generations of tomorrow (4, 5), searching for causes and solutions with others rather than their own groups. Xenophobia is on the rise, and right-wing populism raising pressure to keep out or send away foreigners and migrants is becoming increasingly represented in governmental bodies (6, 7). In short, the challenge of persuading individuals to act cooperatively, and not just when members of their own group are concerned, rather than maximizing short-term self- and in-group interests, has yet to be resolved.

How can this large-scale failure of equity in human behavior be understood? Research on human cooperation shows that the pervasive effect of group membership biasing prosocial behavior in favor of the in-group (1), also referred to as in-group favoritism or parochial cooperation/altruism (8), is at the core of this issue. Individuals have been shown to be more generous toward in-group members (9, 10). This is the case both when group membership is determined by social categorization based on real-life characteristics such as gender, age, nationality, or migration status and even when group membership is determined by trivial criteria [e.g., preference for paintings by Klee or Kandinsky (10–12)] or even by chance [e.g., the outcome of a coin-flip (13, 14)]. Group membership, therefore, is a powerful driver of prosocial behavior (i.e., decisions to benefit others, potentially at a cost to oneself) and parochially directs it toward the in-group, leaving the out-group disadvantaged.

However, behavioral research on in-group favoritism aiming to explain large-scale cooperation failure often implicitly assumes or experimentally interferes with the mental processes involved in constructing a choice (15, 16). In contrast, explicitly studying the mental processes underlying choice behavior promises insights into the actual cognitive processes of searching for and processing choice-relevant information, constructing preferences, and preparing subsequent behavior (17, 18), while minimizing perturbations of the processes under investigation (19). Research on in-group favoritism has made some contributions to testing decision processes underlying parochiality in prosocial behavior (20–23). However, for this research area, as for research on higher-order cognitive processes (24, 25) and beyond (26–29), it has traditionally been a particular challenge to test the generalizability of findings. Research assessing cognition in an unobtrusive, fine-grained manner often requires in-lab data collection with specialized research apparatuses, effectively excluding populations outside of areas with dedicated labs. While decades of behavioral research demonstrating in-group favoritism may well be used to argue for generalizability across time and across situational differences (24, 25, 30–32), the generalizability of cognitive processes underlying discriminatory decision-making remains understudied.

We argue that the key to explaining parochial cooperation lies in understanding both the underlying cognitive processes that drive individuals’ prosocial decision behavior favoring their in-group, as well as macrolevel cultural factors at play in parochial altruism, the big-picture facets of culture and country-level differences influencing (processes of) generosity and discrimination. Combining both levels of analyses, we leverage a recent methodological advance, the development of webcam-based eye-tracking (33). Webcam-based eye-tracking yields sufficient data quality in terms of spatial and temporal resolution, but is more flexible and (cost-) efficient in use compared to traditional lab-based systems (34). Using human research participants’ webcams to measure gaze behavior creates the possibility to reach an unprecedentedly diverse population in terms of cultural and geographic variance for cognitive research, regardless of on-the-ground research facilities, democratizing access to research participation. Eye-tracking provides real-time data on information search and processing and allows inferences about cognitive states through a variety of eye movement metrics which are closely linked to the underlying mechanisms (35, 36). Pairing a utility model of prosocial decision-making in the intergroup context (9) with domain-general models of information processing (37, 38) yields explicit predictions (39) about the relation of individual-level preferences, macro-level factors, and high-resolution gaze behavior.

Here, we leverage webcam-based eye-tracking to study the cognitive steps involved in preparing decisions about whether to act prosocially toward in- and out-group members, expanding existing research to the international context. We report data from a preregistered eye-tracking study (preregistration available via https://osf.io/hnspe, data, materials, and code available on the OSF, materials on GitHub) using webcam-based gaze estimations in incentivized prosocial decision tasks in the intergroup context. This data collection allowed us to bring together gaze and choice data from 1,850 participants in 20 nations, where nations were characterized along preregistered, distinct facets of culture (e.g., individualism vs. collectivism), social institutions (e.g., government effectiveness), and health (e.g., COVID-19 disease burden).

**Fig. 1.**
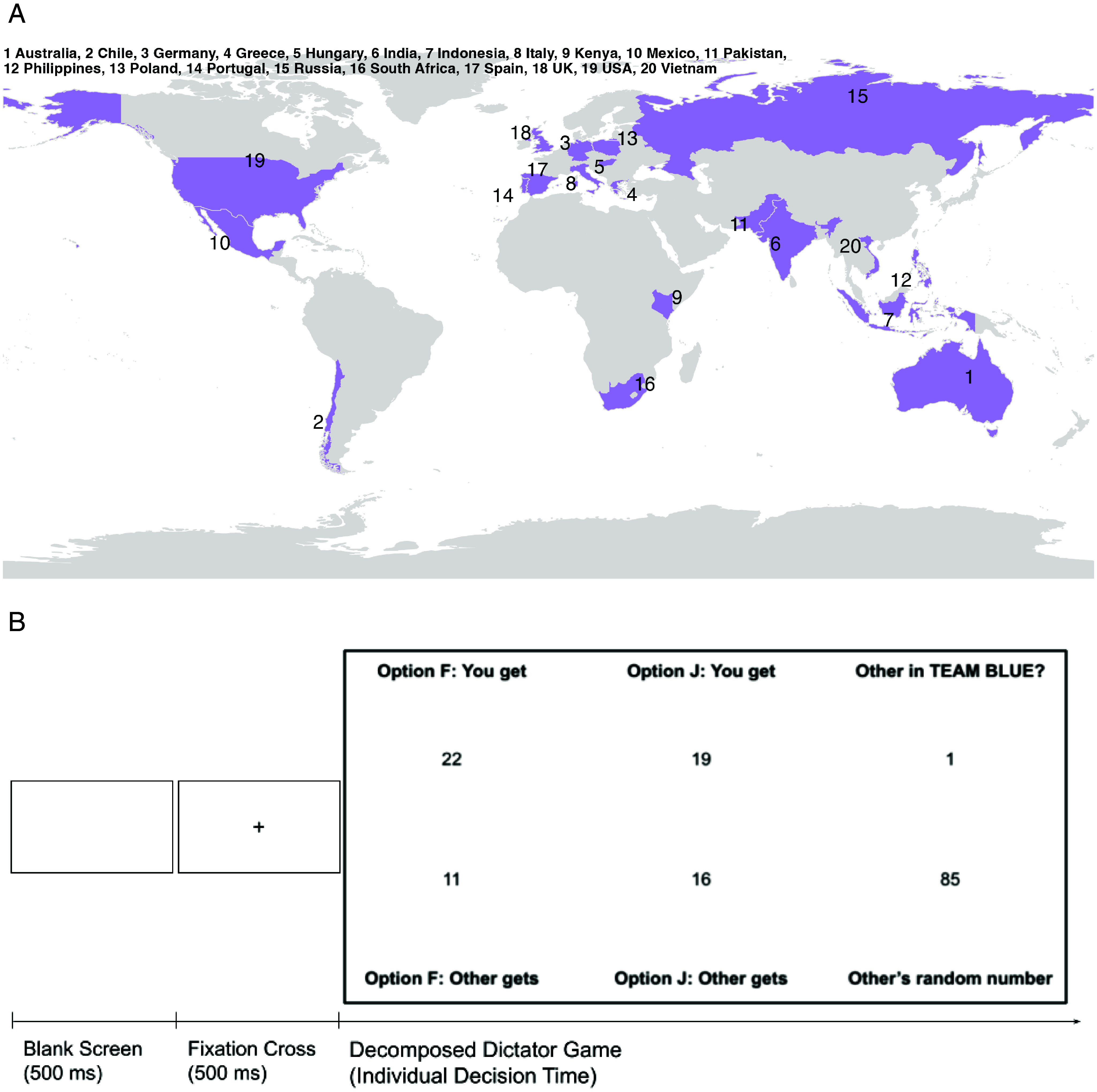
Regions of data collection (Panel *A*) and exemplary trial of the main task (Panel *B*). *Note*. Panel *A* shows the countries in which data were collected (numbered in alphabetical order). Panel *B* demonstrates the trial setup in the main task, during which eye-gaze was recorded. Note that information presented was printed in small font (12 px) and spread out across the display to avoid peripheral legibility. In each trial, a blank screen was presented, followed by a fixation cross in the center of the screen. Subsequently, the main decision screen displayed a decomposed dictator game in a table format. Participants chose between options F and J, neutrally labeled corresponding to the buttons pressed to log in their decision. For each option displayed in one of two columns, they learned how many points they would get and how many the matched player would get (displayed in rows). In the third column, information about the other player was displayed: a random number associated with them (nondiagnostic information) as well as the group membership from the perspective of participants’ own team (same team = 1; different team = 0; diagnostic information). Participants were carefully instructed on how to read the table and completed both control questions and example trials. Columns and rows were counterbalanced between participants. In target trials, one option always represented a selfish choice where participants maximized their own outcomes, while the other option represented a prosocial choice in which they could give up a small proportion of potential earnings to benefit the other player, while still earning more than the other player would.

Departing from prior behavioral research on cross-cultural differences in in-group favoritism using real groups, we assessed discriminatory decisions in minimal group settings. Participants were allocated to groups based on their decisions in a color identification task (40) followed by an incentivized group reinforcement task in which participants competed against an out-group member for a monetary bonus to increase the importance of the in- vs. out-group. They then faced 80 decisions in decomposed dictator games with matched players who belonged to either their in- or the out-group (Fig. 1). In each of the 58 target items, they decided between two options. Each time, if they chose the selfish option, they maximized their own payoff but the matched player would receive only little. If they chose the prosocial option, however, they would earn less than if they had chosen the selfish option, but the payment to the other player would increase as well as the sum of both payments (Fig. 1). During these decisions, we captured gaze positions directed to the screen via participants’ webcams.

## Behavioral In-Group Favoritism Is Prevalent Across the Globe

**Fig. 2.**
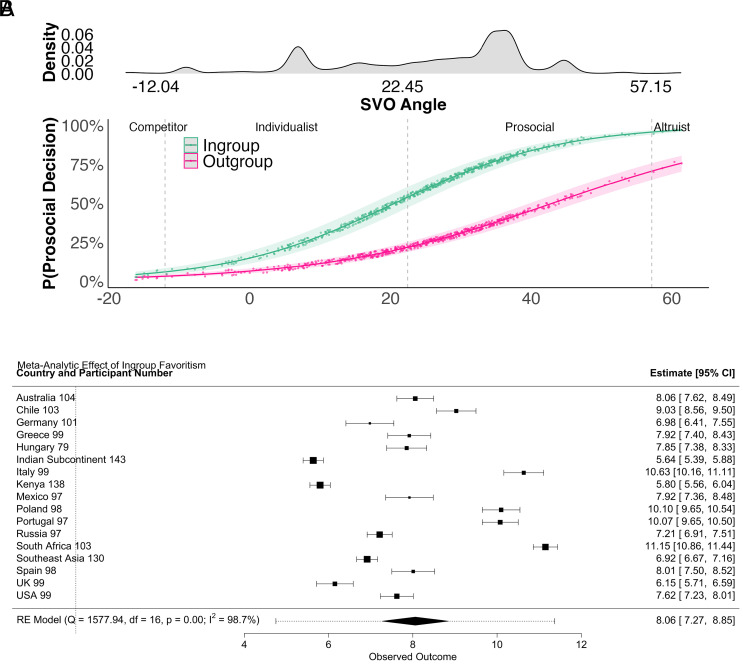
Density plot of SVO distribution and probability of making prosocial decisions depending on the matched player’s group membership and own SVO (Panel *A*), and meta-analytic effect of in-group favoritism (Panel *B*)*. Note*. Panel *A* includes 95% confidence bands, points represent (jittered) predicted values. For a version of Panel *A* including observed data, see *SI Appendix*, Fig. S1. In Panel *B*, countries are ordered alphabetically to aid comparison across figures. Squares represent country-level point estimates, with larger boxes indicating larger effect sizes. Error bars are 95% CI. The pooled effect is indicated by the diamond shape, where the diamonds’ width corresponds to the 95% CI, with a surrounding prediction interval. Data from countries with fewer than 80 observations were pooled to regional groups (Indonesia, Philippines, Vietnam form the Southeast Asia group, India, and Pakistan form the Indian Subcontinent group).

Facing an in- rather than an out-group member increased the odds of making prosocial decisions [OR = 4.57, 95% CI(3.44,6.11), *P* < 0.001, as preregistered, Fig. 2, Panel *A* and *SI Appendix*, Table S1]. Performing a preregistered meta-analysis with random effects models across countries showed that in-group favoritism was present in each country studied, although effect sizes differed substantially (Fig. 2, Panel *B*). Discrimination between in- and out-group members in prosocial decisions was larger for prosocial compared to selfish participants [determined via SVO angle (41), OR= 1.36, 95% CI(1.32,1.41), *P* < 0.001 as preregistered], suggesting the tendency for parochialism was more prevalent among prosocials (Fig. 2, Panel *A*). This interaction effect that prosocials discriminated more between the in- and out-group was also demonstrable in the preregistered meta-analytical assessment across most countries studied, although it reversed in some (exploratory, *SI Appendix*, Fig. S2).

## Societal Differences in Behavioral In-Group Favoritism can be Explained by Country-Level Predictors

Given the considerable variation in in-group favoritism between societies, we sought to identify potential explanations for this country-level heterogeneity (Table 1). Two theoretical perspectives yielded preregistered hypotheses, testing the effect of country-level predictors on in-group favoritism.

**Table 1.** Logistic mixed effects model predicting prosocial decisions from standardized country-level predictors

	Prosocial decision		
Predictors	OR	z	p
Intercept	0.28	−7.22	**<0.001**
Group setting (1 = in-group, 0 = out-group)	4.82	10.77	**<0.001**
Individualism	0.76	−3.50	**<0.001**
Government effectiveness	0.74	−2.80	**0.005**
Religiosity	0.67	−4.40	**<0.001**
Historic disease burden	1.11	1.00	0.319
COVID-19 burden	0.85	−1.96	0.050
English as second language (1 = yes, 0 = no)	0.99	−0.09	0.927
Platform (Prolific = 1, other = 0)	1.36	1.64	0.102
Trial Index	1.00	−1.68	0.093
Group setting × Individualism	1.15	6.08	**<0.001**
Group setting × Government Effectiveness	0.88	−4.15	**<0.001**
Group setting × Religiosity	1.07	2.66	**0.008**
Group setting × Historic Disease Burden	0.70	−12.23	**<0.001**
Group setting × COVID-19 Burden	1.25	10.39	**<0.001**
N _item_number_	58		
N _subject_	1783		
N _country_model_	20		
Observations	1,38,064		
Marginal R^2^/conditional R^2^	0.091/0.616		

*Note.* Test against null model: χ^2^ (11) = 1,043.28, *P* < 0.001. Analysis uses unpooled country predictors.

On the one hand, the Material Security Hypothesis (24, 42, 43) proposes that in societies facing greater uncertainty and instability, individuals seek out closer and more homogenous social support networks. The advantages of such close-knit systems trigger a generalized tendency for in-group favoritism, more so than in societies where efficient institutions provide a solid basis from which risky investments in relationships with out-group members can be afforded. To test the prediction of the Material Security Hypothesis, that in-group favoritism is more pronounced in societies with greater uncertainty, we considered four markers of societal uncertainty: the degree to which both governmental and religious institutions are valued, as well as the historic and burden of disease during the preceding COVID-19 pandemic. As predicted, in countries where the government was characterized as less effective as per the World Bank Governance Indicator (44), discrimination in favor of the in-group was larger. Although we had predicted that decreased importance of religion in everyday life [scores taken from the World Value Survey] (45) would lead to increased in-group favoritism, evidence pointed in the opposite direction: The more important religious institutions were, the larger the extent of in-group-favoring discrimination. Regarding the historic (46) and COVID-19-specific (47) burden of disease, we found that lower disability adjusted life years and a higher total number of confirmed deaths due to COVID-19 reported were related to increased in-group favoritism. These findings supported the preregistered hypotheses that health-related uncertainty (both in terms of a looming history of disease burden, as well as public perceptions of recent medical insecurity following many COVID-19 deaths) would be associated with increased discrimination in favor of the in-group. Overall, evidence suggested that reduced uncertainty would support equal treatment of in- vs. out-group members, with the exception of predictions regarding religion as a social institution. (*SI Appendix*, Fig. S3).

On the other hand, the Cultural Dimensions Theory (48) proposes that cultures can be clustered based on differentiating factors. The individualism-collectivism factor, describing the degree to which social interdependence is favored, has been linked to in-group-favoring discrimination (49): Closer social ties, as promoted in more collectivist cultures, are hypothesized to support increased reputational concerns, leading to increased in-group favoritism [(50), preregistered]. However, results in this study showed an effect in the opposite direction: in-group favoritism increased as country-level individualism rose (*SI Appendix*, Fig. S4). This pattern of results supports the argument that individualistic cultures provide fertile ground for in-group biases because of the increased need and craving for identification with different possible in-groups (42).

In sum, the substantial country-level heterogeneity in the degree to which in-groups were favored and out-groups discriminated against was systematically related to indicators of societal uncertainty, both regarding institutions and health concerns, as well as to individualism as a cultural dimension. However, the directionality of the effects of religiosity and individualism was the opposite of the theoretical predictions.

## Country-Level Heterogeneity in Effects of Processing Effort for and Attention Distributed to In-groups

**Fig. 3.**
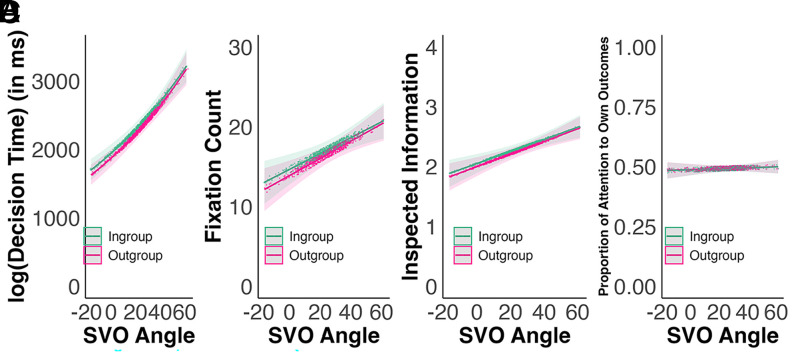
Decision effort [decision time (Panel *A*), fixation count (Panel *B*), and inspected information (Panel *C*)], and attention distribution (Panel *D*) by SVO. *Note*. 95% confidence bands, points represent (jittered) predicted values. For a version of this graph including observed data, see *SI Appendix*, Fig. S5.

**Fig. 4.**
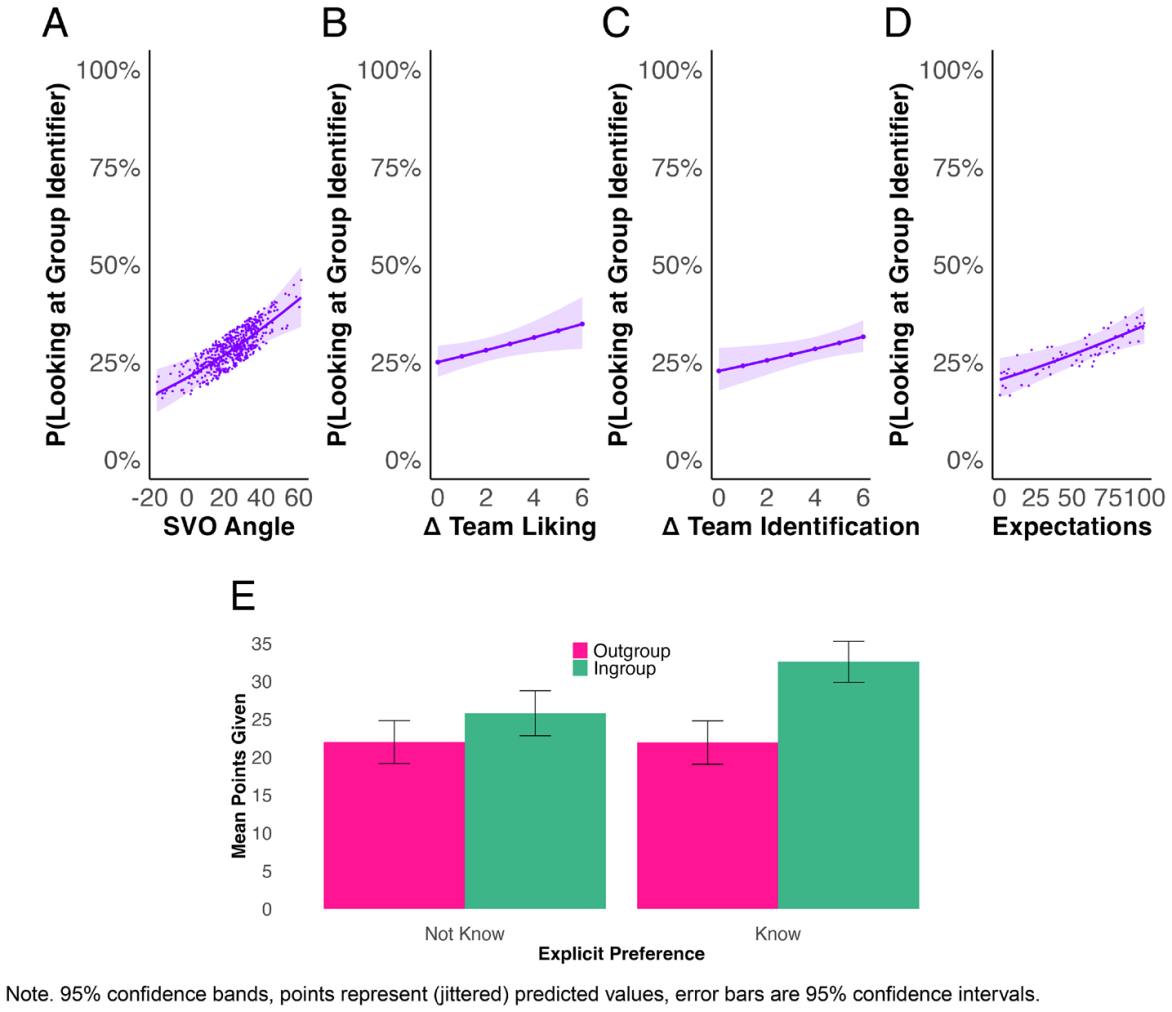
Meta-analytic effects of in-vs. out-group setting on decision time (Panel *A*), fixation counts (Panel *B*), the number of inspected pieces of information (Panel *C*) and the proportion of attention directed to own vs. others’ outcomes (Panel *D*). *Note.* Analyses ran only on trials in which group identifying information was gazed at at least once. Performing these analyses on all trials, even those in which participants remained blind to the group membership of the other player, yielded qualitatively similar results (*SI Appendix*, Fig. S6). Squares represent country-level point estimates, with larger boxes indicating larger effect sizes. Error bars are 95% CI. The pooled effect is indicated by the diamond shape, where the diamonds’ width corresponds to the 95% CI, with a surrounding prediction interval.

Further, we tested cognition about in-group favoritism during prosocial decision-making. We predicted that decision effort, conceptualized in terms of decision time, fixation count, and the number of available pieces of information that were inspected, would be increased when decision makers faced a decision to benefit an in-group member compared to an out-group member (21–23, 41), preregistered). Pooling all data, results consistently demonstrated that facing an in-group member triggered more effortful processing (Fig. 3, Panels *A*–*C* and Table 2). Performing preregistered meta-analyses with random effects models across countries similarly yielded positive, significant meta-analytic effects of in-group favoritism for all three measures of decision effort (Fig. 4, Panels *A*–*C*): On average, decision effort invested was higher when participants faced an in- rather than an out-group member. Yet, at the country level, significant in-group favoritism effects demonstrated substantial between-country heterogeneity, varying in size and directionality (*SI Appendix*, Fig. S6).

**Table 2.** Linear mixed effects models predicting decision effort (log-transformed decision time (in ms), fixation counts, and the number of inspected pieces of information) and attention distribution

	log(decision time)			Fixation count			Inspected information			Attention to own outcomes		
*Predictors*	*β*	*t*	*p*	*β*	*t*	*p*	*β*	*t*	*p*	*β*	*t*	*p*
Intercept	8.20	277.70	**<0.001**	39.55	34.26	**<0.001**	4.00	52.24	**<0.001**	0.51	38.74	**<0.001**
Group setting (1 = in-group, 0 = out-group)	0.04	2.22	**0.026**	1.14	2.08	**0.038**	0.08	3.13	**0.002**	−0.00	−1.24	0.215
SVO angle	0.10	7.91	**<0.001**	2.10	4.17	**<0.001**	0.16	4.80	**<0.001**	0.00	0.26	0.798
English as second language (1 = yes, 0 = no)	0.05	2.13	**0.033**	−1.01	−0.99	0.321	−0.04	−0.61	0.540	0.00	0.39	0.700
Platform (Prolific = 1, other = 0)	0.13	4.70	**<0.001**	−0.82	−0.75	0.453	0.27	3.65	**<0.001**	−0.01	−1.14	0.253
Trial Index	−0.00	−77.32	**<0.001**	−0.10	−59.87	**<0.001**	−0.01	−57.71	**<0.001**	−0.00	−1.04	0.298
Group setting × SVO angle	−0.00	−0.89	0.375	−0.27	−1.64	0.100	−0.02	−1.96	0.051	0.00	0.70	0.484
N	58_item_number_			58_item_number_			58_item_number_			58_item_number_		
	1,446 _subject_			1,446 _subject_			1,446 _subject_			1,298 _subject_		
Observations	41741			41741			41741			37522		
Marginal R^2^/Conditional R^2^	0.095/0.623			0.045/0.592			0.047/0.626			0.001/NA		

*Note.* Tests against null models were significant for effort variables [Model 1: χ^2^ (3) = 66.57, *P* < 0.001; Model 2: χ^2^ (3) = 22.39, *P* < 0.001; Model 3: χ^2^ (3) = 33.37, *P* < 0.001], but not for attention distribution [Model 4: χ^2^ (3) = 2.09, *P* = 0.553]. Analyses ran only on trials in which group identifying information was gazed at at least once. Performing these analyses on all trials, even those in which participants remained blind to the group membership of the other player, yielded no significant main effects of the group setting (*SI Appendix*, Table S2).

Similarly, we assessed preferential attention allocation to information about others’ payoffs (vs. own payoffs) but found no evidence of in-group favoritism in the preregistered pooled analysis (Fig. 3, Panel *D*). As preregistered, we also assessed the effect of in-group favoritism in terms of attention distribution to others’ outcomes meta-analytically (Fig. 4, Panel *D*), showing a net-zero meta-analytic effect, although all country-specific estimates of the in-group favoritism effect were significantly different from zero.

Therefore, while in some countries discrimination between in- and out-group in terms of decision effort and attention distribution favored the in-group, in other countries, the effect was reversed, suggesting that out-group-facing decisions required more effort and sparked more attention to others’ outcomes.

## Individual Preferences Condition Decision Processes During Prosocial Decision-Making

Combining social preferences (51, 52) with attentional drift diffusion models (37, 38), we predicted that individuals’ decision effort would differ as a function of their social preferences (37): When individuals’ prosocial orientation increases, it diminishes the perceived utility differences between selfish and prosocial options as more weight is placed on others’ outcomes compared to decision processes of individualistic decision makers [as derived in (39)]. Because making decisions between options perceived to differ less requires more decision effort (37, 53), more prosocial preferences are associated with increased decision effort (39, 54). Results consistently demonstrated that more prosocial participants invested more effort in informing their choice (Fig. 3, Panels *A*–*C* and Table 2).

We further assumed that utility derived from benefitting an out-group member would be discounted (9, 55) compared to an in-group member. This discounting factor more strongly affects the perceived utility difference and therefore the required processing effort between choosing prosocially or selfishly when decision makers’ preferences are more prosocial. Consequently, we expected an interaction effect demonstrating that more prosocial decision-makers would show larger processing differences in in- vs. out-group cases. In the preregistered pooled analysis, however, there was no evidence for the predicted interaction effect (Fig. 3 and Table 2). Assessing this interaction effect meta-analytically on a country level, however, showed that this net-zero overall effect could be decomposed into individual, significant effects of varying size and directionality depending on the country (*SI Appendix*, Fig. S7).

Although we had further made preregistered predictions about preferential allocation of attention to others’ outcomes (37, 56, 57), the preregistered pooled analyses indicated no evidence that the decision processes of increasingly prosocial individuals had allocated more attention to others’ payoffs (Fig. 3, Panel *D* and Table 2).

## Societal Differences in Cognition About In-Group Favoritism not Explained by Country-Level Predictors

To investigate the differences in cognition about in-group favoritism between countries, we again consulted the Material Security Hypothesis (43) and Cultural Dimensions Theory (48), pairing them with domain-general models of decision processes (37, 38). Similarly to the effects of social preferences on decision effort and attention allocation, we expected that factors associated with greater behavioral discrimination between the in- vs. out-group would also be associated with greater processing differences between in- vs. out-group (preregistered).

Although some of the preregistered country-level variables tested were associated with changes in decision effort or attention distribution (Table 3 and *SI Appendix*, Table S3), these relations were relatively unsystematic. Since the measures for decision effort tend to be highly correlated, we would have expected meaningful effects to generalize across all three variables, but found only sporadic effects. Similarly, using predictors of Material Security and Cultural Dimensions as moderators in exploratory multivariate metaregressions did not significantly explain the variability in effect sizes regarding effort variables [*Q_log(decision time)_* (5) = 6.07, *P* = 0.30, *Q_fixation count_* (5) = 1.55, *P* = 0.91, *Q_inspected information_* (5) = 8.2034, *P* = 0.15] or attention distribution [*Q_attention to own outcomes)_* (5) = 6.09, *P* = 0.30]. In addition, we explored whether SVO distribution (proportion of prosocial and altruistic compared to individualistic and competitive types; range of distribution) within countries explained differences in effect sizes between countries. We found no evidence for such meta-analytic moderating effects regarding decision effort [*Q_log(decision time)_* (2) = 3.54, *P* = 0.17, *Q_fixation count_* (2) = 1.71, *P* = 0.42, *Q_inspected information_* (2) = 2.38, *P* = 0.30], or attention distribution [(*Q_attention to own outcomes)_* (2) = 0.38, *P* = 0.83]).

**Table 3.** Linear mixed effects models predicting decision effort (log-transformed decision time, fixation counts, and the number of inspected pieces of information) and attention distributions

	log(decision time)			Fixation count			Inspected information			Attention to own outcomes		
*Predictors*	*β*	*t*	*p*	*β*	*t*	*p*	*β*	*t*	*p*	*β*	*t*	*p*
Intercept	8.18	112.59	**<0.001**	35.98	12.56	**<0.001**	3.92	27.94	**<0.001**	0.48	24.23	**<0.001**
Group setting (1 = in-group, 0 = out-group)	0.04	2.20	**0.028**	1.12	2.04	**0.041**	0.08	3.04	**0.002**	−0.00	−1.17	0.240
Individualism	0.03	0.85	0.398	0.94	0.71	0.478	0.09	1.28	0.201	−0.00	−0.26	0.795
Government effectiveness	−0.06	−1.18	0.236	−1.19	−0.62	0.533	−0.11	−1.14	0.255	−0.00	−0.32	0.746
Religiosity	0.03	0.89	0.373	0.70	0.50	0.615	0.04	0.54	0.592	0.00	0.27	0.784
Historic disease burden	−0.10	−2.14	**0.032**	0.34	0.18	0.854	−0.09	−1.02	0.306	0.01	0.95	0.341
COVID-19 Burden	−0.06	−1.52	0.129	−0.97	−0.63	0.529	−0.07	−0.88	0.377	−0.01	−0.61	0.544
English as second language (1 = yes, 0 = no) (1 = yes, 0 = no)	0.04	1.13	0.261	0.58	0.46	0.646	0.00	0.06	0.956	0.01	0.75	0.453
Platform (Prolific = 1, other = 0)	0.17	1.90	0.057	2.67	0.76	0.449	0.34	2.03	**0.043**	0.02	0.95	0.341
Trial Index	−0.00	−77.28	**<0.001**	−0.10	−59.83	**<0.001**	−0.01	−57.68	**<0.001**	−0.00	−1.02	0.308
Group setting × Individualism	0.00	0.40	0.692	−0.16	−0.67	0.504	0.03	2.14	**0.033**	0.00	0.34	0.735
Group setting × Government Effectiveness	−0.01	−1.27	0.202	−0.04	−0.11	0.911	−0.01	−0.37	0.711	−0.00	−0.40	0.688
Group setting × Religiosity	−0.02	−3.45	**0.001**	−0.48	−1.68	0.092	0.00	0.25	0.801	−0.01	−1.08	0.281
Group setting × Historic Disease Burden	−0.00	−0.64	0.522	0.12	0.40	0.687	−0.02	−0.87	0.386	−0.00	−0.53	0.597
Group setting × COVID-19 Burden	0.00	0.43	0.665	0.18	0.82	0.411	0.01	1.05	0.296	−0.00	−0.80	0.426
N	58_item_number_			58_item_number_			58_item_number_			58_item_number_		
	1,446_subject_			1,446_subject_			1,446_subject_			1,298_subject_		
	17_country_model_			17_country_model_			17_country_model_			17_country_model_		
Observations	41741			41741			41741			37522		
Marginal R^2^/conditional R^2^	0.079/0.628			0.043/0.595			0.041/0.628			0.003/0.353		

*Note.* Tests against null models: Model 1: χ^2^ (11) = 46.37, *P* < 0.001; Model 2: χ^2^ (11) = 14.55, *P* = 0.204; Model 3: χ^2^ (11) = 27.94, *P* = 0.003; Model 4: χ^2^ (11) = 10.96, *P* = 0.446. Analyses ran only on trials in which group identifying information was gazed at at least once, with unpooled country predictors.

## Colorblindness: Probability of Looking Up Group Membership

Despite the overall effect of behavioral discrimination between in- and out-group, look-up rates of other’s group membership information, an indicator of visual (in)attention to group colors, were low. Even when relaxing the temporal definition criterion for fixations and including gazes shorter than 50ms, in the majority of trials (53.49%), group membership information was gazed at not even once, violating the assumption that costless but relevant information would rationally be accessed (56). However, participants visually attended to information revealing others’ group membership significantly more often than to the matched players’ random number (33.28% of trials, exploratory Wilcoxon signed-rank test with continuity correction: *V* = 1026481, *P* < 0.001, *r* = 0.39 (medium-sized effect)). Therefore, they distinguished between diagnostic (group-indicating) and nondiagnostic (random) information.

**Fig. 5.**
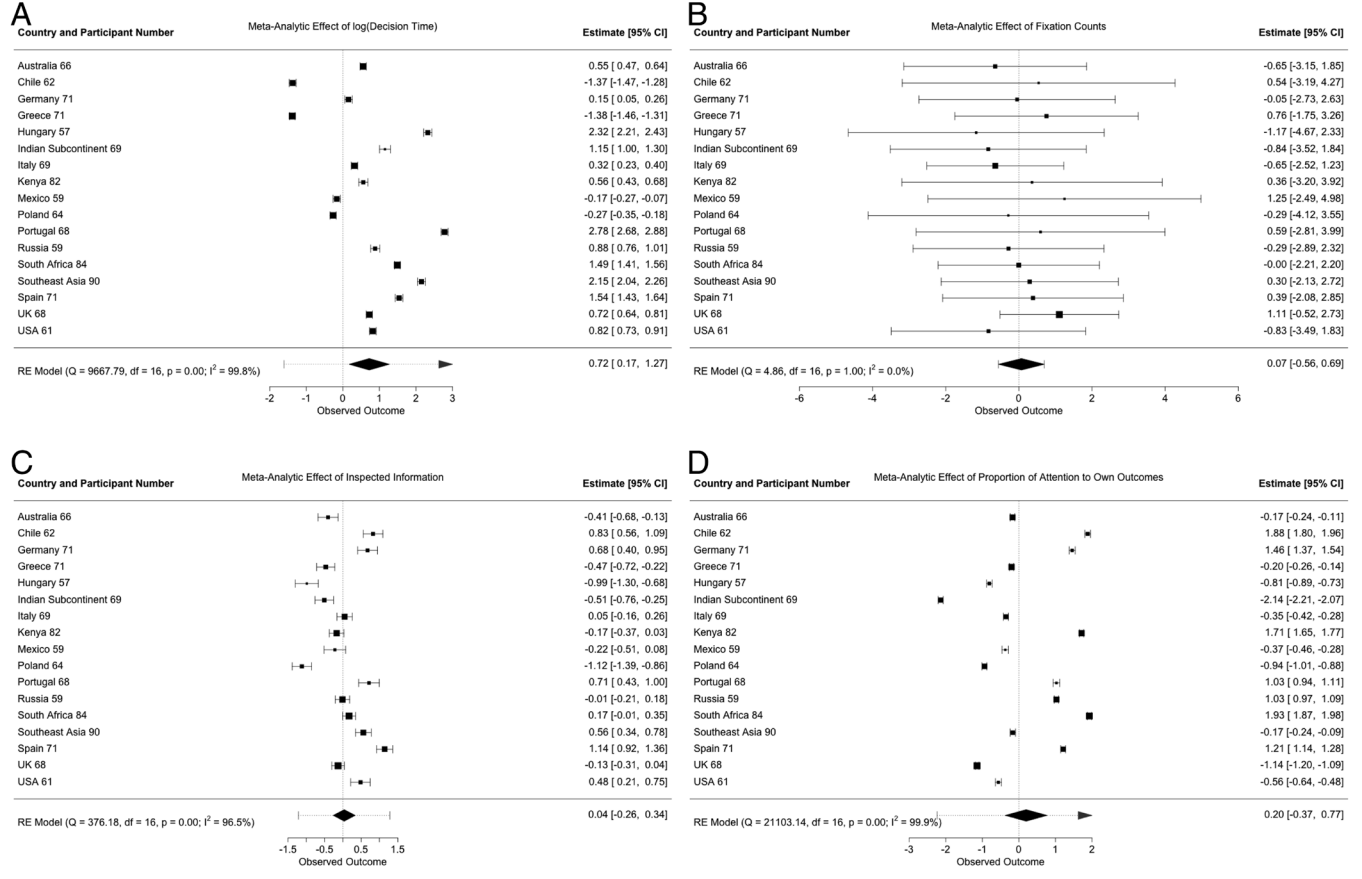
Visual inattention toward group identifying information depending on SVO (Panel *A*), difference in liking (Panel *B*), and identifying with (Panel *C*) the own vs. other team, as well as expectations of look-ups (Panel *D*); and in-group favoritism depending on explicit preference to learn matched players’ group membership (Panel *E*). *Note*. 95% confidence bands, points represent (jittered) predicted values, error bars are 95% CI. For a version of Panels *A*–*D* including observed data, see *SI Appendix*, Fig. S8.

Participants’ individual-level characteristics predicted whether they would remain colorblind: Increasingly prosocial preferences, better liking of and higher identification with their team compared to the out-group, as well as increasing expectations that others would look up group membership predicted higher odds of looking up group identifying information oneself (Fig. 5, Panels *A*–*D* and *SI Appendix*, Table S4, preregistered). Society-level characteristics associated with the Material Security Hypothesis and Cultural Dimensions Theory, however, did not (Table 4, preregistered).

**Table 4.** Logistic mixed effects models predicting visual attention to group membership

	Looking up group		
*Predictors*	*OR*	*z*	*p*
Intercept	0.24	−5.34	**<0.001**
SVO angle	1.26	3.83	**<0.001**
Δ team identification	1.08	2.24	**0.025**
Δ team attitude and liking	1.09	2.39	**0.017**
Expectations	1.01	3.31	**0.001**
English as second language (1 = yes, 0 = no)	0.94	−0.44	0.662
Platform (Prolific = 1, other = 0)	1.31	1.02	0.308
Trial Index	1.00	−20.38	**<0.001**
Individualism	1.10	0.92	0.360
Government effectiveness	0.80	−1.50	0.133
Religiosity	1.02	0.12	0.904
Historic disease burden	0.86	1.01	0.312
COVID-19 Burden	1.09	0.76	0.448
N_item_number_	58		
N_subject_	1,630		
N_country_model_	20		
Observations	90,359		
Marginal R^2^/conditional R^2^	0.034/0.613		

*Note*: Test against null model: χ^2^ (9) = 57.16, *P* < 0.001. Analysis uses unpooled country predictors.

## Colorblindness: Explicit Preferences to Remain Blind to Group Membership

To assess explicit preferences for colorblindness, at the end of the experiment session, we asked participants to play one more incentivized dictator game. This time, they were asked explicitly if they wanted to find out the team color of the player they were matched with. The majority of players (54.79%) explicitly preferred to unveil the matched player’s group membership. Explicit preferences to find out others’ group membership predicted visual attention to group membership information (*OR* = 1.91, *z* = 5.45, *P* < 0.001, *SI Appendix*, Table S7, preregistered). Colorblindness preferences conditioned whether participants discriminated between the in- and out-group in this game (*β* = 6.96, *t* = 2.41, *P* = 0.02, see Fig. 5, Panel *E* and *SI Appendix*, Table S6, exploratory), where participants were informed about the matched player’s group membership regardless of their preference. Those who wanted to remain colorblind but were informed about the matched players’ group membership anyway did not significantly discriminate against out-group compared to in-group partners (*β* = 3.576, *t* = 2.08, *P* = 0.09). However, those who wanted to learn about the matched player’s group membership were significantly more generous toward their in-group, discriminating against the out-group by giving about 10% less to them than to in-group members (*β* = 10.54, *t* = 2.00, *P* < 0.001).

## Discussion

In-group favoritism is a robust and enduring phenomenon. In this study, we provide further evidence that in-group favoritism exists across cultures (1, 17): Discrimination in prosocial decision-making in favor of the in-group was demonstrated in every country studied, yielding a large overall effect in the pooled analysis (OR = 4.58). This was the case even though we studied discrimination in a minimal group setting, departing from the prior literature that largely demonstrated the cross-cultural stability of in-group favoritism using real groups.

In addition, we provide evidence about individual- and country-level correlates of discrimination. Results aligned with prior evidence that decision-makers’ orientation toward prosocial values was often associated with increased discrimination (58, 59), although cross-cultural variations suggested that the relationship between discrimination and SVO would reverse in some nations. This finding adds a further perspective on the debate on whether parochial altruism is primarily driven by prosocial decision makers (59, 60): Whether increased prosocial orientation exacerbates or moderates discrimination may also depend on decision makers’ cultural background.

Findings further suggested that societal uncertainty and a culture of individualism were largely associated with increased in-group-favoring discrimination. In contrast with the expectations of the Material Security Hypothesis, increased importance of religion as an indicator of stable national institutions was associated with more discrimination (see (61) for a similar result showing increased discrimination under stable institutions). For government ineffectiveness, as well as historic and COVID-19-specific disease burden as further indicators of societal uncertainty, results indicated that higher uncertainty was related to increased discrimination.

Going beyond the observation of choice behavior, this study provides evidence that culture modifies cognitive processes involved in informing and preparing discriminatory decision-making. Enabled by a dataset that captured gaze data from a large and diverse participant group, evidence showed that in-group decisions were more effortfully prepared and that individual preferences for prosociality were related to increased visual search effort, corroborating prior findings (39). Importantly, the directionality of the interaction effect of in-group vs. out-group setting and prosociality differed between countries: While in some countries studied, prosocials favored in-group members by investing more effort in informing their decision when an in-group member was concerned, in other countries studied, the effect reversed. Attempting to link individuals’ gaze behavior to the preregistered society-level differences of interest, however, could not explain when more discrimination in terms of decision effort invested would take place. These findings therefore indicate that cognitive processes underlying discriminatory decision-making differ across cultures, with systematic explanations for these cross-cultural differences still outstanding.

Similarly, individual-level predictors explained decision-makers’ remaining colorblind toward (i.e., visually not attending to) others’ group membership, where society-level predictors did not. Colorblindness was linked to selfish preferences (low SVO), lending further support to the idea that more prosocial decision makers more readily discriminate (58, 59)—and visually look up the information necessary to do so. Individuals appeared motivated to find out others’ group membership to benefit their team, since increased team identification and liking were linked with increased probabilities of visually attending to group identifying information. Finally, holding expectations that others would uncover group information led individuals to look up this information themselves, potentially because they (correctly) expected that others would use this information to bias their decisions (62).

Whether decision-makers explicitly chose to seek out information that would identify the matched player as an in- or out-group member conditioned their subsequent discrimination: While those who preferred not to remain colorblind discriminated in favor of their in-group, there was no evidence of discrimination among those who preferred to remain colorblind. Overall, the degree to which participants remained colorblind—whether in terms of explicitly avoiding or in terms of visual non-attendance to group identifying information—was relatively high. This suggests that assuming widespread interest in information necessary to bias decisions when average behavioral effects show discrimination might be misguided. Instead, a minority preferring not to remain colorblind, characterized by a large difference in attitudes toward the in- vs. out-group, may be driving these behavioral effects. This finding further highlights the importance of studying cognitive processes underlying decision behavior instead of merely making assumptions about these processes (16).

In sum, these findings challenge assumptions of cross-cultural generalizability of prior findings relating to behavioral in-group favoritism and cognitive processes underlying discrimination. Attempting to explain cross-cultural variation in behavioral in-group favoritism based on the Material Security Hypothesis and the Cultural Dimensions Theory yielded mostly successful predictions, but attempting to apply these theories to the high-resolution process data could not explain cross-cultural differences in how decisions were constructed. Therefore, a demonstrable need for theoretical developments in social cognition that explicitly model not only choice-level but also process-level predictions has emerged (63, 64). Certainly, testing such theories with appropriately diverse samples will be an important step for future research toward understanding the processes underlying human decision behavior, beyond parochial altruism. Such research is facilitated by technological advances broadening access to research participation, although limitations regarding demonstrations of causality necessarily remain. Since cultural or sociodemographic factors cannot be experimentally manipulated, unobserved, or uncontrolled variation stemming from interindividual or between-country homogeneity (e.g., relative (personal) wealth, social status, language) complicate the interpretability of results (see exploratory analysis on the role of speaking English as a second language and personal migratory history on discrimination, *SI Appendix*, Fig. S9 and Table S8). Future research in this intercultural context should carefully study the role of factors such as instruction language and payoff schemes as they may substantially influence both cognition and behavior (65). Not only providing standardized instructions in English but adapting to participants’ native languages may trigger culturally afforded values, norms, or behaviors (66). Similarly, adapting payoffs to personal wealth or country purchasing power may reveal differential processing of resulting high- and low-stakes environments (67). Both factors are unobserved in this study, limiting the conclusions that can be drawn about the meaning of intercultural variation demonstrated here. Triangulating the influence of such factors of human diversity is something to be embraced, challenging traditionally generalizing assumptions.

With an eye to applications to practice, this study highlights the critical role of cultural considerations and demonstrates the potential for insights from cross-cultural research in social cognition to inform and transform global policymaking. On the global scale, policymaking often affects groups of different cultures, requiring cooperation to reach joint goals: Challenges necessitating culturally informed policies cover a wide range of issues from fair resource allocation, health policies, and social integration policies. Failure to take into consideration differences in how cooperative decisions between groups will be constructed dooms the chances that one-size-fits-all interventions will be particularly effective. Although nontailored interventions may operate as intended on average, institutions in some countries might find such policies to backfire. Particularly for institutional design and policy interventions on the global level, the recognition that between-group variations may determine effectiveness leads to a clear need for advancing cross-culturally informed decision-making. In addition, smaller-scale instances requiring intergroup cooperation, such as nationwide intervention roll-outs or intraorganizational change processes, may also benefit from explicitly considering potential between-group differences in behavior and cognition to ensure optimal fit between the assumed mechanism of the intervention and the actual circumstances in the groups addressed.

## Method

Participants (*N_collected_* = 1850, *N_after exclusions_* = 1792, *M_age_* = 26.22, *SD_age_* = 4.78, 777 female, 30 diverse) were recruited online in spring 2023. The study was conducted under ethics block approval of the Max Planck Ethics Committee to the Bonn DecisionLab. Participants received the equivalent of £3.5 paid in the Platform (Prolific = 1, other = 0)-specific currency as a fixed participation fee, with a £1.45 variable payoff depending on their and others’ decisions and performance. The study followed a 2 (group: in- vs. out-group, within subjects) × 20 (country, between subjects) x SVO (continuous, between subjects) mixed design.

Participants gave informed consent, completed the SVO slider task (41), were allocated to their respective in- and out-groups based on a color identification task and completed an incentivized reaction time task to increase the perceived importance and relevance of the groups. In the incentivized *main task*, participants faced 80 trials (58 target and 2 filler items) of decomposed dictator games, deciding between two options to allocate points between themselves and a matched player (Fig. 1, Panel *B*). Each time, we presented one selfish and one prosocial option, for which participants learned their potential earnings and the potential earnings of the matched player. In addition, each trial contained two pieces of information about the other player: whether they were an in- or out-group member (indicated as 1 or 0) and a random number assigned to this person (between 10 and 89). For details regarding the counterbalanced decision screen, on which information was presented in small font and spaced out across the display to avoid peripheral legibility, see Fig. 1, Panel *B*.

In a final incentivized *dictator game*, participants decided how to allocate 100 points between themselves and another player. Before making their decision, participants indicated whether they would like to learn the other players’ group membership (“Would you like to know which team the other participant belongs to?” yes or no). Regardless of the answer, they were randomly presented either with an in- or out-group member, or with a participant with unknown group membership.

Additional tasks included a manipulation check, and assessments of group identification and cultural orientations. Demographics, English language status, and country of residence data were also collected. An in-depth description of the procedure and materials is available in the *SI Appendix*.

## Supplementary Material

Appendix 01 (PDF)

## Data Availability

Anonymized raw data without personal identifiers, as well as code and a link to the software used have been deposited in OSF (https://osf.io/8zqjf/) (68).
