# Supplementary material for: Cognitive processes of ingroup favoritism across 20 countries: An eye-tracking investigation of culture, behavior, and cognition
Source: Proc Natl Acad Sci U S A. 2025 Aug 5;122(32):e2417456122. doi: 10.1073/pnas.2417456122 (PMC12358900; doi:10.1073/pnas.2417456122)
Supplement: Supplementary file 1 — Appendix 01 (PDF) [file pnas.2417456122.sapp.pdf]

Supplementary Material
for
Cognitive processes of ingroup favoritism across 20 countries: An eye-tracking investigation of culture, behavior and cognition

Methods **3**
Participants and Design 3
Procedure 4
References **7**
Figure S1 **8**
Probability of making prosocial decisions depending on the matched player's group membership and own SVO, with observed data 8
Figure S2 **9**
Meta-analytic effect of ingroup favoritism by SVO interaction 9
Figure S3 **10**
Ingroup favoritism depending on uncertainty in societal institutions (Panels A and B) and society-level health indicators (Panels C and D) 10
Figure S4 **11**
Ingroup favoritism depending on society-level differences in individualism 11
Figure S5 **12**
Decision effort (decision time (Panel A), fixation count (Panel B), and inspected information (Panel C)), and attention distribution (Panel D) by SVO, with observed data 12
Figure S6 **13**
All trials: Meta-analytic effects of in- vs. outgroup setting on decision time (Panel A), fixation counts (Panel B), the number of inspected pieces of information (Panel C) and the proportion of attention directed to own vs. others' outcomes (Panel D) 13
Figure S7 **14**
All trials: Meta-analytic effects of interaction of in- vs. outgroup setting and SVO on decision time (Panel A), fixation counts (Panel B), the number of inspected pieces of information (Panel C) and the proportion of attention directed to own vs. others' outcomes (Panel D) 14
Figure S8 **15**
Visual inattention towards group identifying information depending on SVO (Panel A), difference in liking (Panel B) and identifying with (Panel C) the own vs. other team, as well as expectations of look-ups (Panel D), with observed data 15
Figure S9 **16**
Probability of making prosocial decisions depending on the matched player's group membership and self-reported status speaking English as second language (0 = English native speaker, 1 = speaker of English as second language; Panel A), as well as migratory history (0 = no migratory history, 1 = spent the majority of their life until turning 18 in a country different than their current country of residence; Panel B). 16
Table S1 **17**
Logistic mixed effects model predicting the odds of making prosocial decisions 17

Table S2 **18**

Linear mixed effects models predicting decision effort (log-transformed decision time (in ms), fixation counts and the number of inspected pieces of information) and attention distribution 18

Table S3 **19**

Linear mixed effects models predicting decision effort (log-transformed decision time (in ms), fixation counts and the number of inspected pieces of information) and attention distribution 19

Table S4 **20**

Logistic mixed effects models predicting visual attention to group membership 20

Table S5 **21**

Logistic mixed effects models predicting visual attention to group membership 21

Table S6 **22**

Linear regressions predicting points given in the final dictator game from players' explicit preference to learn the matched player's group membership, the matched players displayed group membership, and the interaction thereof 22

Table S7 **23**

Linear mixed effects models predicting decision effort (log-transformed decision time (in ms), fixation counts and the number of inspected pieces of information) and attention distribution with trial-level predicted preferences 23

Table S8 **24**

Logistic mixed effects models predicting the probability of making a prosocial decision 24

Table S9 **25**

Overview of hypotheses and results regarding decision behavior 25

Table S10 **26**

Overview of hypotheses and results regarding gaze behavior 26

Methods

Participants and Design

Participants ($N_{collected}$ = 1850, $N_{after\ exclusions}$ = 1792, M_{age} = 26.22, SD_{age} = 4.78, 777 female, 30 diverse) were recruited via online participant platforms (Prolific, MTURK, Toloka, recruitment platform used as control variable in analyses reported) in spring 2023. We recruited participants aiming to reach 100 completed responses each from the following 17 countries: Australia, Chile, Germany, Greece, Hungary, India, Italy, Mexico, Philippines, Poland, Portugal, South Africa, Spain, UK, USA, Afghanistan, and Bangladesh. However, because we received no responses from Afghanistan and Bangladesh on MTURK within the first two days of data collection, we instead collected data via Toloka from Pakistan, Kenya, Russia, Indonesia and Vietnam. Country selections were based on availability via the online platforms used, and aimed to maximize between-country differences in individualism scores and to spread data collection out geographically. We anticipated that where per-country sample size would fall below 80, data could be pooled regionally and set a maximum recruitment time of 2 weeks per country.

Participants received the equivalent of £3.5 paid in the platform-specific currency as a fixed participation fee. In addition, participants could receive up to £1.45 variable payouts depending on their and others' decisions and performance in the tasks outlined below. The fixed payment and possible additional payment for each task was described in the local currency. Note that we chose not to adjust average payments by country purchasing power to ensure that a fair wage would be paid to all participants regardless of their location, in line with the requirements of conducting experiments using the platform Prolific. While stake-size could partially contribute to between-country heterogeneity, personal wealth differences could partially explain inter-individual heterogeneity. To address these issues, analyses focused on the probability of

choosing prosocially (not on amounts given), toward the in- vs. outgroup (i.e., relative, not absolute giving).

The study lasted about 20 min, and participants were eligible to take part if they had a working webcam, were between 18 and 35 years old and spoke English well because the experiment was presented in English for all participants. Note that we deliberately chose to present the materials in English throughout to avoid differences in the visual displays of the stimuli between languages. We prioritized capturing top-down processes of attention allocation, and sought to avoid bottom-up differences that might introduce noise. Since English language skills may have differed between participants, we included a control variable of whether the participant indicated that English was their second language in the analyses presented.

The study followed a 2 (group: in- vs. outgroup, within subjects) x 20 (country, between subjects) x SVO (continuous, between subjects) mixed design.

Procedure

The experiment was presented in an online application implemented via jsPsych (*1*) using the webgazer (*2*) plugin. Participants indicated informed consent and gave permission to use their webcam. They then indicated their age and gender, and completed the 6-item version of the SVO slider task (*3*).

In the *group allocation task*, participants then saw 8 trials showing color bars, 2 of which were clearly blue, 2 clearly green, and 4 mixed blue-green tones (*4*), in random order. For each bar they indicated whether they perceived it as green or blue. The color they indicated more often determined their allocation to Team Green or Team Blue, and participants were then informed of their group membership.

In the *group reinforcement task* implemented to increase the perceived importance and relevance of the groups, participants completed a 12-trial incentivized reaction time competition. Their task was to press the correct key in response to a yellow, purple or blue-green star appearing in random order as quickly as possible (*5*). Correct responses earned 10 points, while false responses lost 10 points. Participants learned that they would compete against a player from the other team and that they could win 100 bonus points for completing the task faster than the other player. Whichever team had more points in the end would earn a bonus payment of the equivalent of £0.55. At the end of the task, participants received feedback about their response time and hit rate, but whether they won would only be calculated after the study had taken place.

In the subsequent *main task*, participants faced 80 trials (58 target and 2 filler items) of decomposed dictator games, deciding between two options to allocate points between themselves and a matched player (Figure 1, Panel B). Each time, we presented one selfish and one prosocial option, for which participants learned their potential earnings and the potential earnings of the matched player. In each trial, participants' own potential outcomes were higher than those of the matched player. They were informed that one randomly drawn decision would be randomly selected to be paid out for them, where they would randomly either be in the role of the dictator determining the outcome for themselves and another player, or in the role of the receiver who would receive the outcome another dictator's decision. 100 points were convertible to the equivalent of £0.30.

In addition, each trial contained two pieces of information about the other player: whether they were an in- or outgroup member (indicated as 1 or 0) and a random number assigned to this person (between 10 and 89). Participants completed three checks for understanding, received instructions for eye-tracking, and underwent a 9-point calibration procedure, followed by a

9-point validation procedure. Thereafter, participants were reminded about the main task, and completed four practice trials. Before moving on to the main task, a calibration (9 points) and validation (4 points) procedure was run, which was repeated again before trials 12, 24, 36, and 48. Participants completed all 80 trials of the main task, during which eye-data was recorded. For details regarding the counterbalanced decision screen, on which information was presented in small font and spaced out across the display to avoid peripheral legibility (see Figure 1, Panel B).

Participants then faced a final *dictator game* in which they decided how to allocate 100 points convertible to the equivalent of £0.30 between themselves and another player. Before making their decision, participants indicated whether they would like to learn the other players' group membership (“Would you like to know which team the other participant belongs to?” yes or no). Regardless of the answer, they were randomly presented either with an in- or outgroup member, or with a participant with unknown group membership. They then decided how many points to allocate to this person, while keeping the remainder to themselves.

Further, we elicited expectations of preferential treatment from ingroup members in the final dictator game (yes vs. no). Next, we elicit expectations about others' behavior in this final dictator game. Participants indicated how many of 100 participants in this study they thought wanted to know the other players' group membership, would give more points to participants from the same team and would expect to get more points from participants from the same team. In a manipulation check participants then indicated which team they belonged to.

To elicit identification with both in- and outgroup, participants complete an adapted version of the Inclusion of Other in the Self Scale(*6*). Then, they indicated the extent to which they saw themselves as a member of and liked the in-and outgroup, respectively, on a 7-point Likert scale (1 = not at all, 7 = totally). Participants then complete an 8-item shortened version of the horizontal and vertical individualism and collectivism scale(*7*), where they answer on a 9-point scale (1 = never to 9 = always).

Finally, we assessed whether participants wore glasses during the study (yes or no), whether English was their native language (yes or no), in which country they spent the most time before turning 18 and in which country they currently lived. The response to the latter question was used to assign participants' country for the data analyses reported here.

References

1. J. R. de Leeuw, jsPsych: a JavaScript library for creating behavioral experiments in a Web browser. *Behav. Res. Methods* **47**, 1–12 (2015).
2. A. Papoutsaki, P. Sangkloy, J. Laskey, N. Daskalova, J. Huang, J. Hays, "WebGazer: Scalable Webcam Eye Tracking Using User Interactions" in *IJCAI* (2016).
3. R. O. Murphy, K. A. Ackermann, M. J. J. Handgraaf, Measuring social value orientation. *Judgm. Decis. Mak.* **6**, 771–781 (2011).
4. B. Simon, R. Brown, Perceived intragroup homogeneity in minority-majority contexts. *J. Pers. Soc. Psychol.* **53**, 703–711 (1987).
5. M. J. Nissen, P. Bullemer, Attentional requirements of learning: Evidence from performance measures. *Cognit. Psychol.* **19**, 1–32 (1987).
6. A. Aron, E. N. Aron, D. Smollan, Inclusion of Other in the Self Scale and the structure of interpersonal closeness. *J. Pers. Soc. Psychol.* **63**, 596–612 (1992).
7. H. C. Triandis, M. J. Gelfand, Converging measurement of horizontal and vertical individualism and collectivism. *J. Pers. Soc. Psychol.* **74**, 118–128 (1998).

Figure S1

Probability of making prosocial decisions depending on the matched player's group membership and own SVO, with observed data

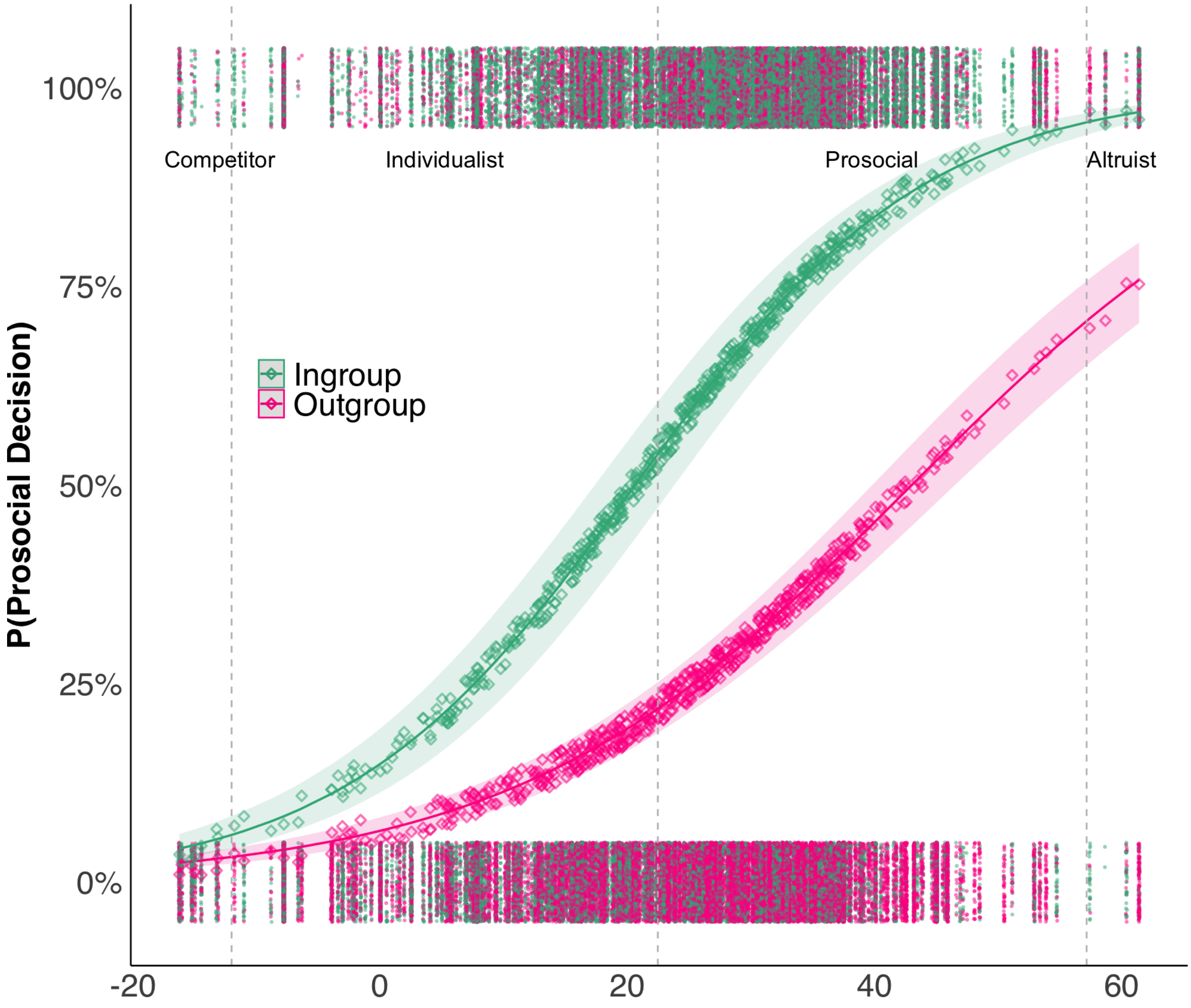

Note. 95% confidence bands, diamonds represent (jittered) predicted values, dots are (jittered) observed data.

Figure S2

Meta-analytic effect of ingroup favoritism by SVO interaction

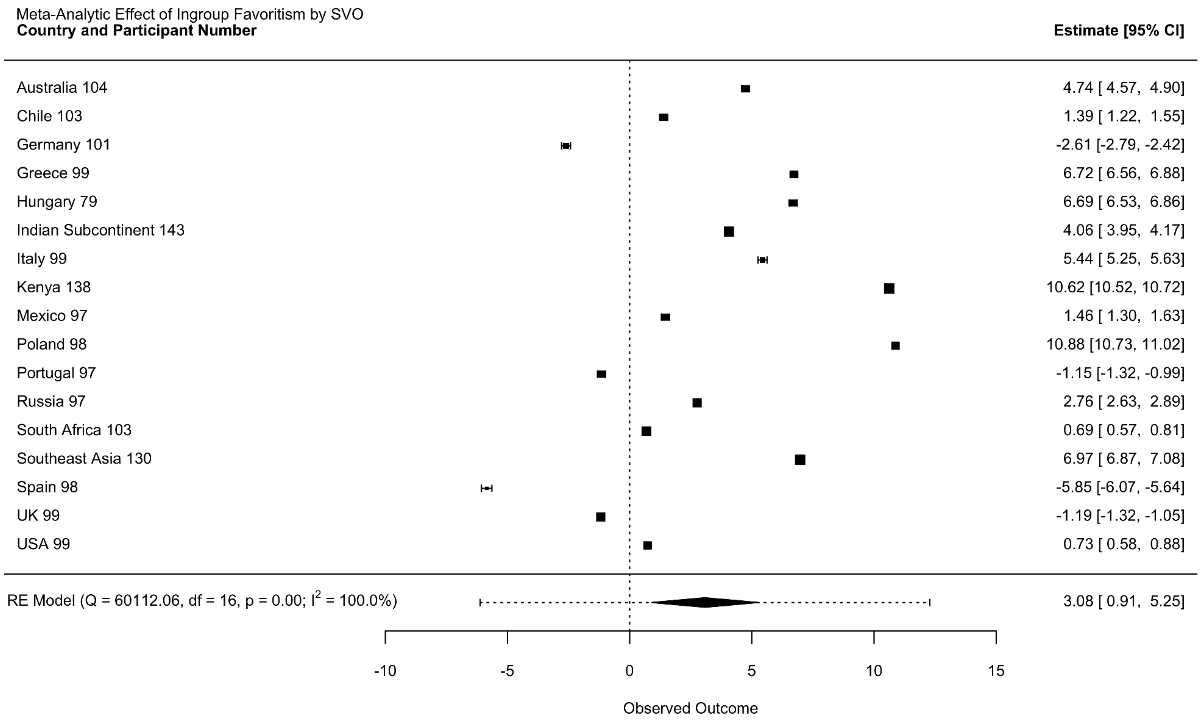

Note. Squares represent country-level point estimates, with larger boxes indicating larger effect sizes. Error bars are 95% confidence intervals. The pooled effect is indicated by the diamond shape, where the diamonds' width corresponds to the 95% confidence interval, with a surrounding prediction interval.

Figure S3

Ingroup favoritism depending on uncertainty in societal institutions (Panels A and B) and society-level health indicators (Panels C and D)

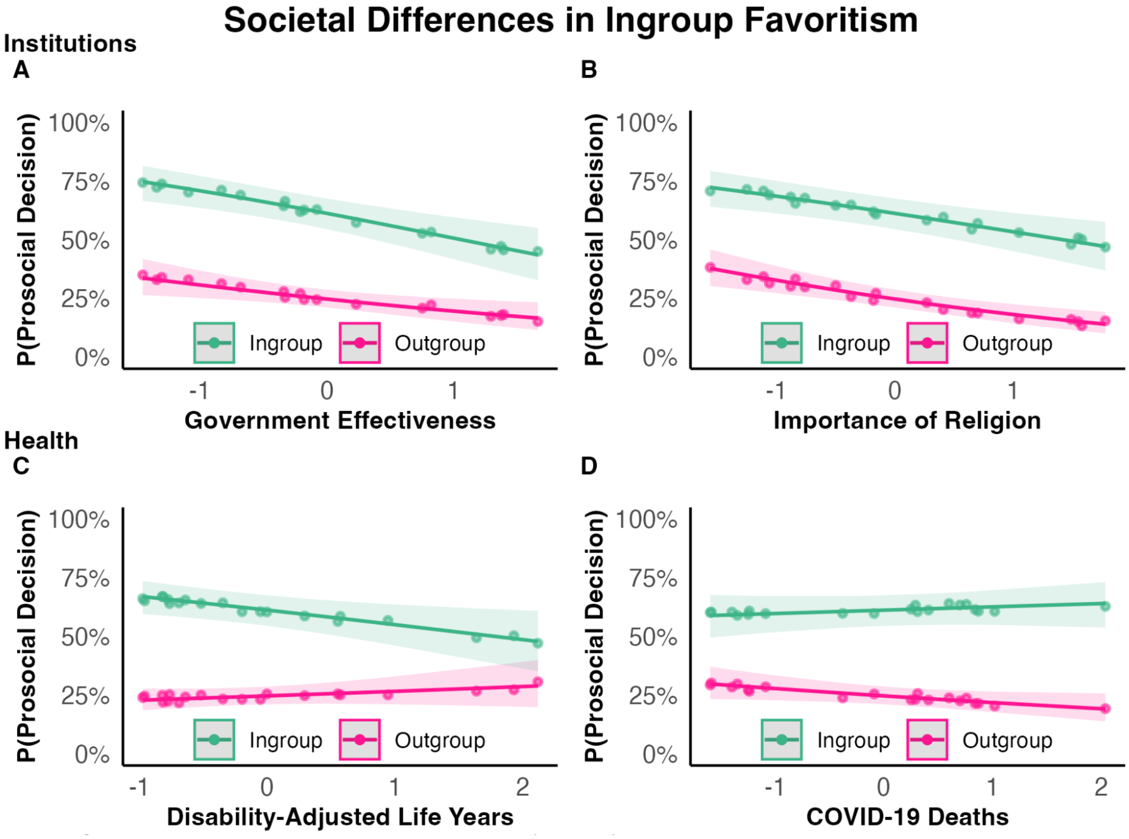

Note. 95% confidence bands, points represent (jittered) predicted values, predictors are standardized.

Figure S4

Ingroup favoritism depending on society-level differences in individualism

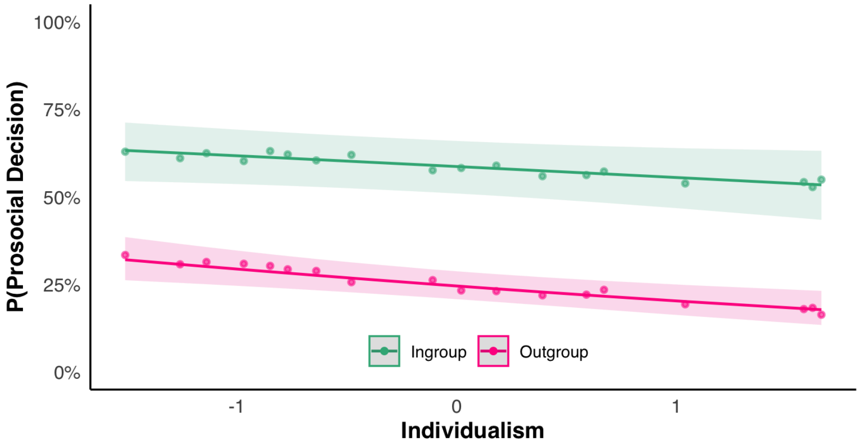

Note. 95% confidence bands, points represent (jittered) predicted values, predictor is standardized.

Figure S5

Decision effort (decision time (Panel A), fixation count (Panel B), and inspected information (Panel C)), and attention distribution (Panel D) by SVO, with observed data

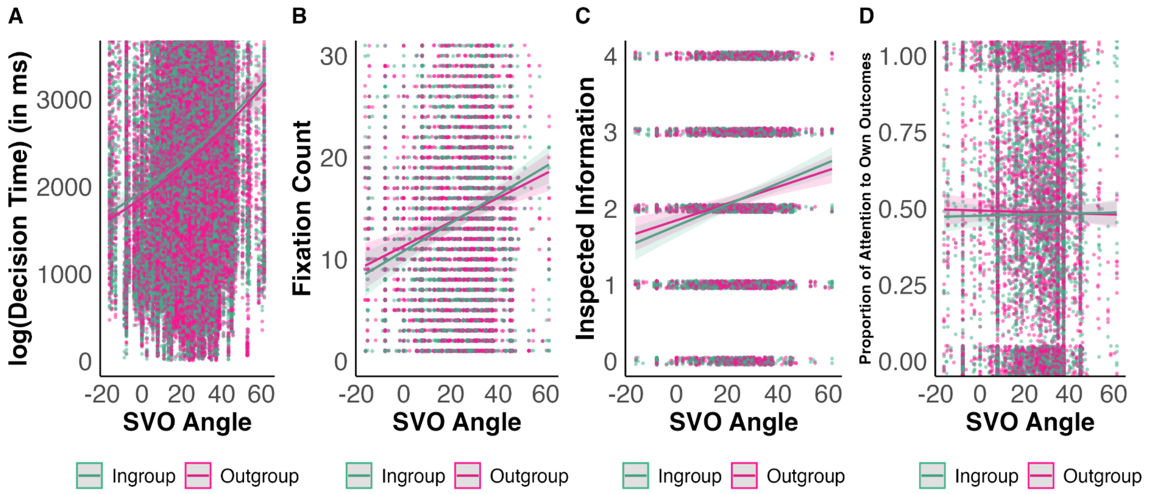

Note. 95% confidence bands, points are (jittered) observed data.

Figure S6

All trials: Meta-analytic effects of in- vs. outgroup setting on decision time (Panel A), fixation counts (Panel B), the number of inspected pieces of information (Panel C) and the proportion of attention directed to own vs. others' outcomes (Panel D)

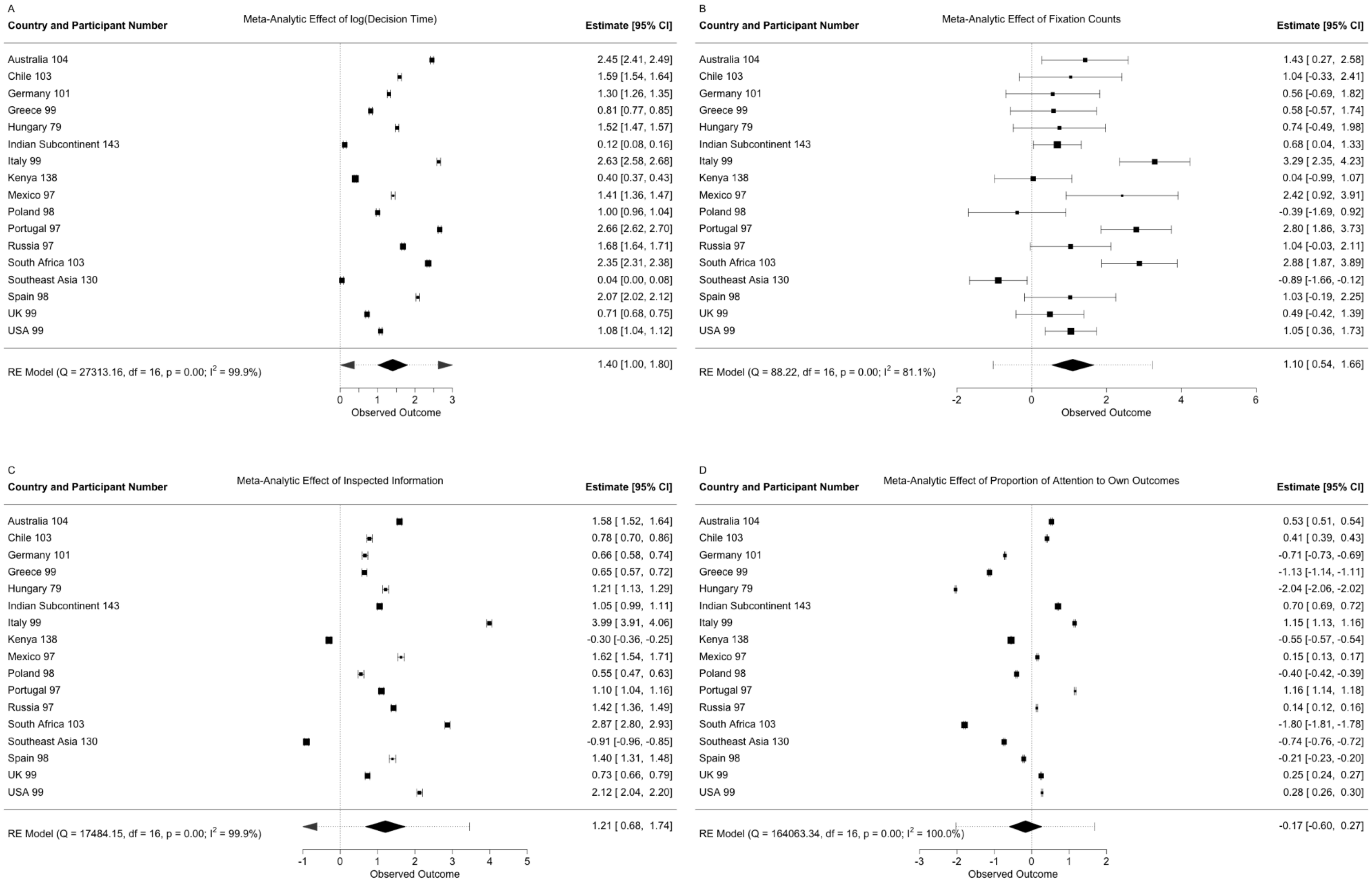

Note. Analyses include all trials, even those in which participants remained blind to the group membership of the other player. Squares represent country-level point estimates, with larger boxes indicating larger effect sizes. Error bars are 95% confidence intervals. The pooled effect is indicated by the diamond shape, where the diamonds' width corresponds to the 95% confidence interval, with a surrounding prediction interval.

Figure S7

All trials: Meta-analytic effects of interaction of in- vs. outgroup setting and SVO on decision time (Panel A), fixation counts (Panel B), the number of inspected pieces of information (Panel C) and the proportion of attention directed to own vs. others' outcomes (Panel D)

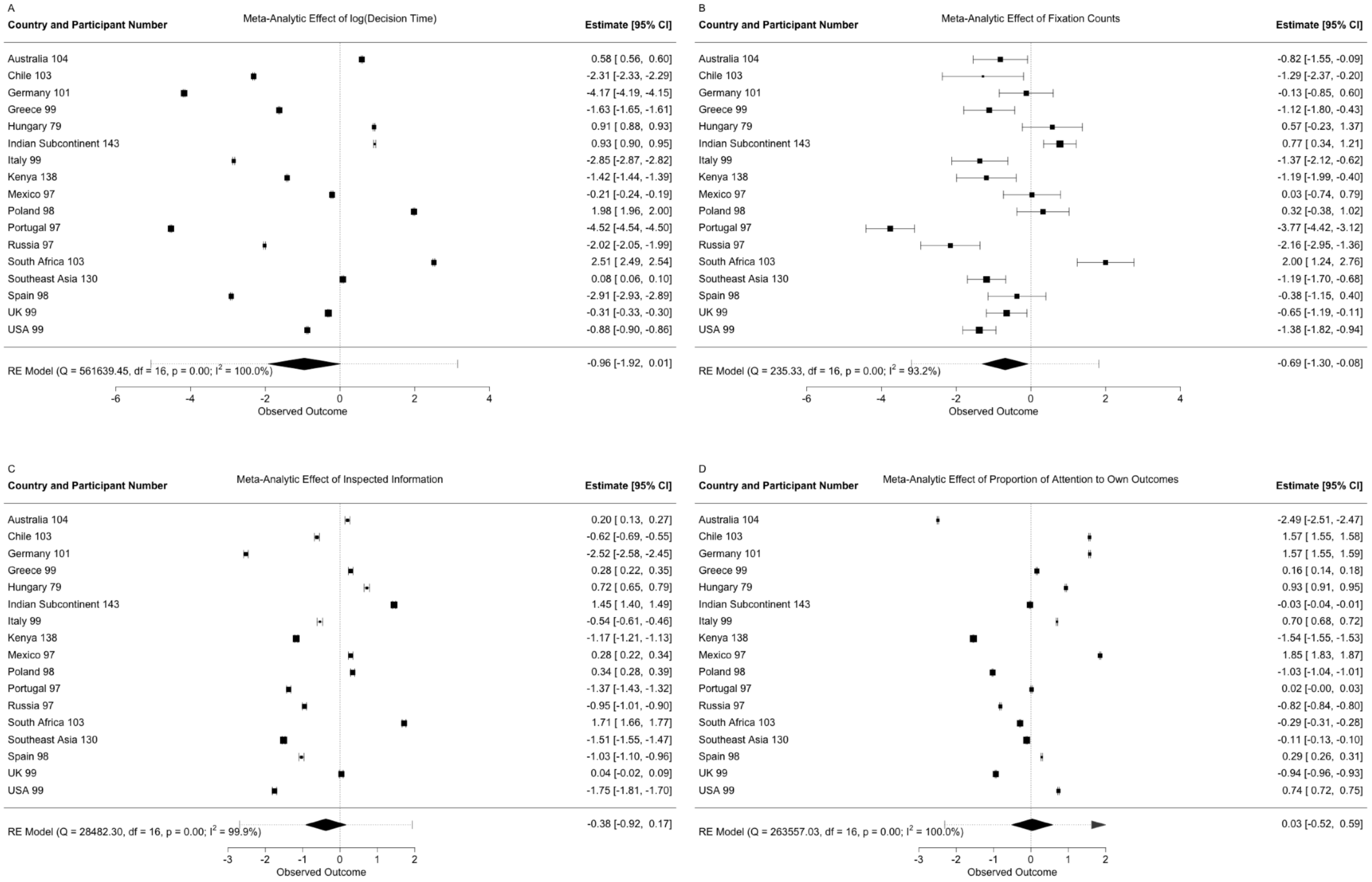

Note. Analyses include all trials, even those in which participants remained blind to the group membership of the other player. Squares represent country-level point estimates, with larger boxes indicating larger effect sizes. Error bars are 95% confidence intervals. The pooled effect is indicated by the diamond shape, where the diamonds' width corresponds to the 95% confidence interval, with a surrounding prediction interval.

Figure S8

Visual inattention towards group identifying information depending on SVO (Panel A), difference in liking (Panel B) and identifying with (Panel C) the own vs. other team, as well as expectations of look-ups (Panel D), with observed data

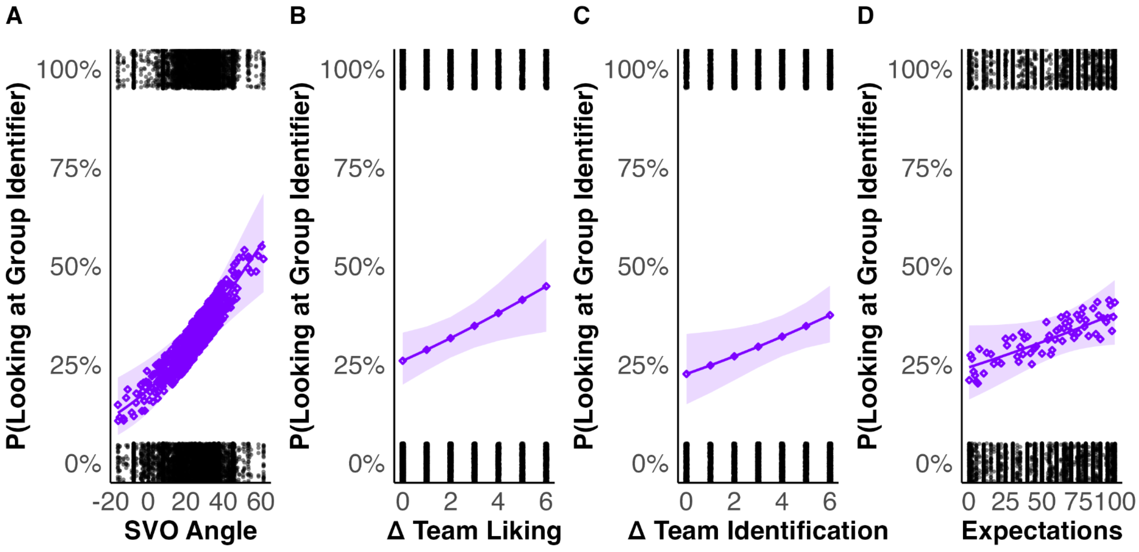

Note. 95% confidence bands, diamonds represent (jittered) predicted values, points are (jittered) observed data.

Figure S9

Probability of making prosocial decisions depending on the matched player's group membership and self-reported status speaking English as second language (0 = English native speaker, 1 = speaker of English as second language; Panel A), as well as migratory history (0 = no migratory history, 1 = spent the majority of their life until turning 18 in a country different than their current country of residence; Panel B).

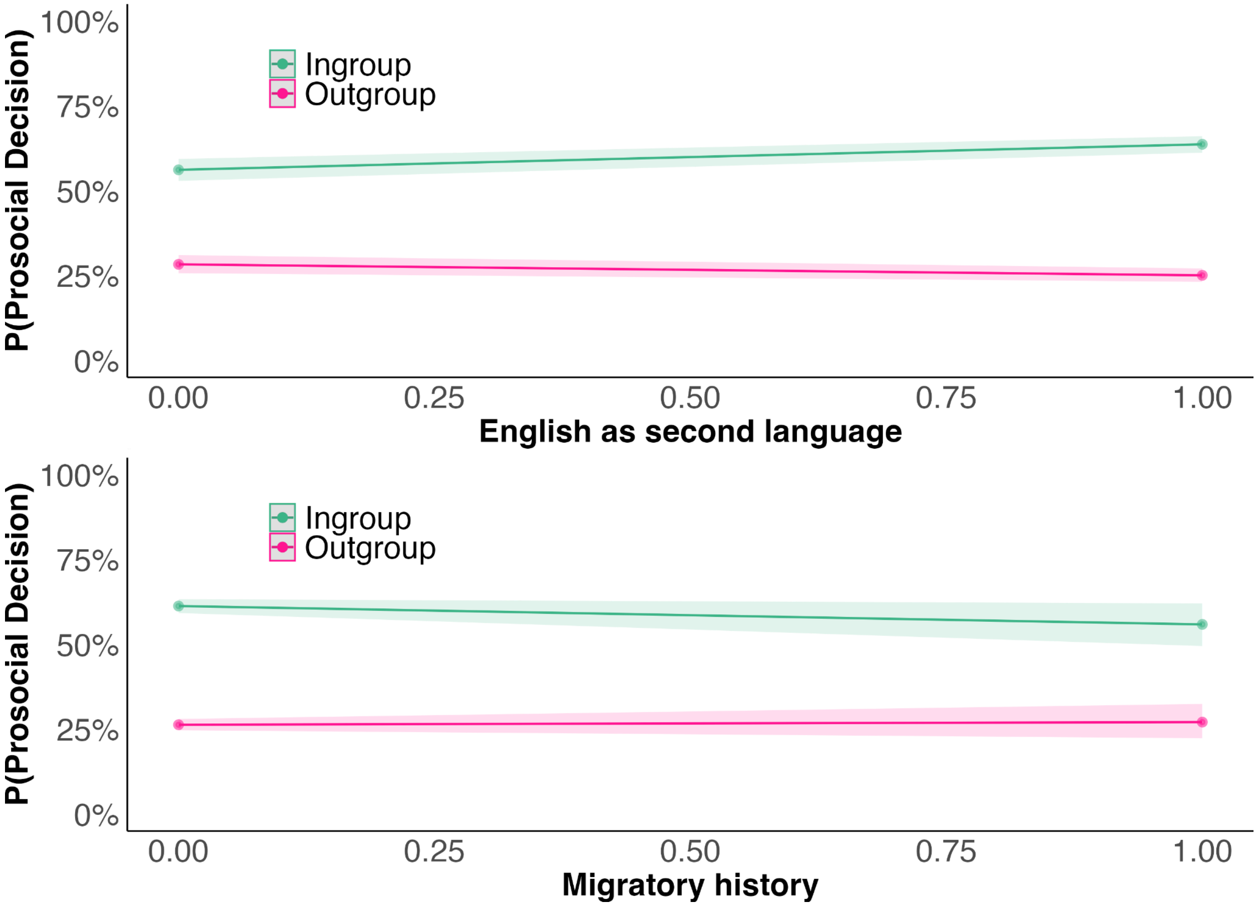

Note. 95% confidence bands, dots represent predicted values.

Table S1

Logistic mixed effects model predicting the odds of making prosocial decisions

	**Prosocial Decision**		
Predictors	*OR*	*z*	*p*
Intercept	0.38	-7.90	**<0.001**
Group setting (1 = ingroup, 0 = outgroup)	4.57	10.33	**<0.001**
SVO angle	2.41	19.87	**<0.001**
English as second language (1 = yes, 0 = no)	1.08	0.89	0.373
Platform (Prolific = 1, other = 0)	0.87	-1.45	0.147
Trial Index	1.00	-1.67	0.095
Group setting × SVO angle	1.37	19.64	**<0.001**
N_{item_number}	58		
$N_{subject}$	1783		
Observations	138064		
Marginal R^2 / Conditional R^2	0.201 / 0.604		

Note. Test against null model: $\chi^2(3) = 949.17$, $p < 0.001$.

Table S2

Linear mixed effects models predicting decision effort (log-transformed decision time (in ms), fixation counts and the number of inspected pieces of information) and attention distribution

	**log(Decision Time)**			**Fixation Count**			**Inspected Information**			**Attention to own outcomes**		
Predictors	*β*	*t*	*p*	*β*	*t*	*p*	*β*	*t*	*p*	*β*	*t*	*p*
Intercept	8.01	249.24	**<0.001**	28.41	30.75	**<0.001**	2.95	38.21	**<0.001**	0.51	43.54	**<0.001**
Group setting (1 = ingroup, 0 = outgroup)	0.03	1.51	0.132	0.56	1.43	0.152	0.04	1.93	0.054	-0.00	-0.35	0.723
SVO angle	0.12	9.15	**<0.001**	1.52	3.78	**<0.001**	0.15	4.31	**<0.001**	0.00	0.52	0.603
English as second language (1 = yes, 0 = no)	0.08	3.09	**0.002**	-0.61	-0.74	0.457	-0.02	-0.35	0.724	-0.00	-0.13	0.893
Platform (Prolific = 1, other = 0)	0.34	11.80	**<0.001**	0.81	0.92	0.358	0.29	3.89	**<0.001**	-0.01	-1.06	0.288
Trial Index	-0.00	-142.79	**<0.001**	-0.07	-86.60	**<0.001**	-0.01	-80.03	**<0.001**	-0.00	-2.97	**0.003**
Group setting × SVO angle	-0.01	-2.09	**0.037**	-0.12	-1.40	0.160	-0.01	-0.89	0.376	0.00	0.08	0.933
N	$58_{\text{item number}}$ 1783_{subject}			$58_{\text{item number}}$ 1722_{subject}			$58_{\text{item number}}$ 1722_{subject}			$58_{\text{item number}}$ 1625_{subject}		
Observations	138064			95477			95477			82300		
Marginal R^2 / Conditional R^2	0.129 / 0.624			0.035 / 0.632			0.037 / 0.668			0.000 / 0.330		

Note. Effects calculated across all trials, even those in which group membership was not gazed at. Tests against null models: Model 1: $\chi^2(3) = 85.74$, p < <0.001; Model 2: $\chi^2(3) = 17.41$, p < 0.001; Model 3: $\chi^2(3) = 22.45$, p <0.001; Model 4: $\chi^2(3) = 0.44$, p = 0.932.

Table S3

Linear mixed effects models predicting decision effort (log-transformed decision time (in ms), fixation counts and the number of inspected pieces of information) and attention distribution

	**log(Decision Time)**			**Fixation Count**			**Inspected Information**			**Attention to own outcomes**		
Predictors	*β*	*t*	*p*	*β*	*t*	*p*	*β*	*t*	*p*	*β*	*t*	*p*
Intercept	8.04	54.19	**<0.001**	25.15	10.77	**<0.001**	2.86	15.97	**<0.001**	0.49	24.42	**<0.001**
Group setting (1 = ingroup, 0 = outgroup)	0.03	1.51	0.132	0.57	1.47	0.142	0.04	1.98	**0.047**	-0.00	-0.30	0.762
Individualism	0.01	0.09	0.929	0.17	0.14	0.885	0.06	0.69	0.491	-0.00	-0.07	0.947
Government Effectiveness	-0.08	-0.73	0.467	-1.54	-0.94	0.347	-0.14	-1.11	0.268	-0.00	-0.09	0.926
Religiosity	0.02	0.24	0.811	-0.57	-0.43	0.669	-0.03	-0.25	0.803	0.00	0.17	0.861
Historic Disease Burden	-0.15	-1.45	0.146	0.35	0.22	0.825	-0.06	-0.50	0.620	0.01	1.03	0.304
COVID19 Burden	-0.04	-0.50	0.619	-0.82	-0.63	0.529	-0.04	-0.44	0.661	-0.01	-0.65	0.517
English as second language (1 = yes, 0 = no)	0.03	0.81	0.416	0.14	0.14	0.888	0.01	0.11	0.914	0.00	0.35	0.728
Platform (Prolific = 1, other = 0)	0.35	1.78	0.075	4.45	1.49	0.136	0.38	1.70	0.089	0.02	0.85	0.396
Trial Index	-0.00	-142.79	**<0.001**	-0.07	-86.59	**<0.001**	-0.01	-80.03	**<0.001**	-0.00	-2.96	**0.003**
Group setting × Individualism	0.00	0.88	0.381	0.11	0.88	0.381	0.03	3.18	**0.001**	-0.00	-0.16	0.874
Group setting × Government Effectiveness	-0.01	-1.62	0.104	-0.30	-1.66	0.097	-0.03	-2.03	**0.042**	-0.00	-0.66	0.510
Group setting × Religiosity	-0.01	-2.82	**0.005**	-0.25	-1.66	0.098	-0.01	-0.57	0.571	-0.00	-0.19	0.853
Group setting × Historic Disease Burden	-0.01	-1.27	0.205	0.01	0.06	0.949	-0.00	-0.35	0.728	-0.01	-1.38	0.166
Group setting × COVID19 Burden	-0.00	-0.38	0.705	0.11	0.87	0.382	0.01	1.07	0.285	-0.00	-1.22	0.224
N	$58_{\text{item number}}$<br>1783_{subject}<br>$20_{\text{country model}}$			$58_{\text{item number}}$<br>1722_{subject}<br>$20_{\text{country model}}$			$58_{\text{item number}}$<br>1722_{subject}<br>$20_{\text{country model}}$			$58_{\text{item number}}$<br>1625_{subject}<br>$20_{\text{country model}}$		
Observations	138064			95477			95477			82300		
Marginal R^2 / Conditional R^2	0.116 / 0.640			0.034 / 0.635			0.032 / 0.671			0.002 / 0.331		

Note. Effects calculated across all trials, even those in which group membership was not gazed at; with unpooled country predictors. Tests against null models: Model 1: $\chi^2(11) = 31.91$, $p < 0.001$; Model 2: $\chi^2(11) = 14.28$, $p = 0.218$; Model 3: $\chi^2(11) = 25.17$, $p = 0.009$; Model 4: $\chi^2(11) = 8.80$, $p = 0.641$.

Table S4

Logistic mixed effects models predicting visual attention to group membership

	**Looking up Group**		
Predictors	OR	z	p
Intercept	0.20	-7.45	**<0.001**
SVO angle	1.25	3.79	**<0.001**
Δ Team Identification	1.08	2.23	**0.026**
Δ Team Attitude & Liking	1.08	2.26	**0.024**
Expectations	1.01	3.23	**0.001**
English as second language (1 = yes, 0 = no)	1.05	0.43	0.669
Platform (Prolific = 1, other = 0)	1.51	3.11	**0.002**
Trial Index	1.00	-20.38	**<0.001**
N_{item_number}	58		
$N_{subject}$	1630		
Observations	90359		
Marginal R^2 / Conditional R^2	0.031 / 0.614		

Note. Test against null model: $\chi^2(4) = 51.60$, $p < 0.001$

Table S5

Logistic mixed effects models predicting visual attention to group membership

	**Looking up Group**		
Predictors	*OR*	*z*	*p*
Intercept	0.32	-7.40	**<0.001**
Explicit preference to look	1.91	5.45	**<0.001**
English as second language (1 = yes, 0 = no)	1.11	0.87	0.382
Platform (Prolific = 1, other = 0)	1.70	4.14	**<0.001**
Trial Index	1.00	-20.39	**<0.001**
N_{item_number}	58		
$N_{subject}$	1630		
Observations	90359		
Marginal R^2 / Conditional R^2	0.052 / NA		

Note. Test against null model: $\chi^2(1) = 28.92$, $p < 0.001$

Table S6

Linear regressions predicting points given in the final dictator game from players' explicit preference to learn the matched player's group membership, the matched players displayed group membership, and the interaction thereof

	**Full Model: Points Given**			**NOT Unveil: Points Given**			**Unveil: Points Given**		
Predictors	*β*	*t*	*p*	*β*	*t*	*p*	*β*	*t*	*p*
Intercept	21.99	14.40	**<0.001**	21.99	14.86	**<0.001**	21.92	15.17	**<0.001**
Unveil Group	-0.07	-0.03	0.974						
Ingroup	3.79	1.77	0.078	3.79	1.82	0.069	10.62	5.32	**<0.001**
Unveil Group × Ingroup	6.84	2.36	**0.019**						
Observations	1175			531			644		
R^2 / R^2 adjusted	0.032 / 0.030			0.006 / 0.004			0.042 / 0.041		

Table S7

Linear mixed effects models predicting decision effort (log-transformed decision time (in ms), fixation counts and the number of inspected pieces of information) and attention distribution with trial-level predicted preferences

	**log(Decision Time)**			**Fixation Count**			**Inspected Information**			**Attention to own outcomes**		
Predictors	*β*	*t*	*p*	*β*	*t*	*p*	*β*	*t*	*p*	*β*	*t*	*p*
Intercept	7.96	240.33	**<0.001**	27.60	29.10	**<0.001**	2.87	36.36	**<0.001**	0.51	42.30	**<0.001**
Group setting (1 = ingroup, 0 = outgroup)	-0.01	-0.49	0.627	-0.01	-0.03	0.977	-0.02	-0.62	0.537	0.00	0.25	0.803
SVO angle	0.11	7.85	**<0.001**	1.28	3.13	**0.002**	0.12	3.56	**<0.001**	0.00	0.63	0.526
Predicted preference	0.15	8.37	**<0.001**	2.34	4.09	**<0.001**	0.23	5.22	**<0.001**	-0.01	-0.71	0.480
English as second language (1 = yes, 0 = no)	0.08	3.04	**0.002**	-0.64	-0.78	0.436	-0.03	-0.39	0.695	-0.00	-0.13	0.899
Platform (Prolific = 1, other = 0)	0.35	11.95	**<0.001**	0.85	0.97	0.334	0.30	3.95	**<0.001**	-0.01	-1.07	0.284
Trial Index	-0.00	-142.57	**<0.001**	-0.07	-86.51	**<0.001**	-0.01	-79.92	**<0.001**	-0.00	-2.98	**0.003**
Group setting × SVO angle	-0.02	-5.15	**<0.001**	-0.26	-2.86	**0.004**	-0.02	-2.79	**0.005**	0.00	0.26	0.793
N	58 item number<br>1783 subject			58 item number<br>1722 subject			58 item number<br>1722 subject			58 item number<br>1625 subject		
Observations	138064			95477			95477			82300		
Marginal R^2 / Conditional R^2	0.132 / 0.625			0.036 / 0.631			0.038 / 0.668			0.000 / 0.329		

Note. Tests against null models, Model 1: $\chi^2(3) = 84.46$, $p < 0.001$; Model 2: $\chi^2(3) = 17.30$, $p < 0.001$; Model 3: $\chi^2(3) = 19.77$, $p < 0.001$; Model 4: $\chi^2(3) = 0.54$, $p = 0.910$. Predicted preferences are predicted probabilities of prosocial decisions from group setting, SVO angle, English as second language, platform, trial number, accounting for item-level and individual variability, obtained via a mixed-effects logistic regression.

Table S8

Logistic mixed effects models predicting the probability of making a prosocial decision

	**Prosocial Decision**			**Prosocial Decision**		
Predictors	*OR*	*z*	*p*	*OR*	*z*	*p*
(Intercept)	0.48	-10.71	**<0.001**	0.44	-18.15	**<0.001**
Group setting (1 = ingroup, 0 = outgroup)	3.24	51.51	**<0.001**	4.43	95.35	**<0.001**
English as second language (1 = yes, 0 = no)	0.85	-1.92	0.055			
Migratory history (1 = yes, 0 = no)				1.04	0.29	0.773
SVO angle	2.39	20.34	**<0.001**	2.35	20.17	**<0.001**
Item	0.99	-15.94	**<0.001**	0.99	-15.95	**<0.001**
Group setting × English as second language	1.61	15.97	**<0.001**			
Group setting × Migratory history				0.77	-5.57	**<0.001**
Group setting × SVO angle	1.27	15.04	**<0.001**	1.30	16.93	**<0.001**
$N_{subject}$	1783			1783		
Observations	138064			138064		
Marginal R^2 / Conditional R^2	0.201 / 0.574			0.199 / 0.572		

Note. Test against null model: Model 1: $\chi^2(4) = 11598.43$, $p < 0.001$; Model 2: $\chi^2(4) = 11376.52$, $p < 0.001$

Table S9

Overview of hypotheses and results regarding decision behavior

#	Analysis Type	Hypothesis	Pre-registered	Result as Expected	Reported in
1a	Pooled	Facing an ingroup vs. outgroup member increases odds of making prosocial decisions	Yes	Yes	Table S1
1b	Meta-Analytic	Facing an ingroup vs. outgroup member increases odds of making prosocial decisions	Yes	Yes	Table S1
2	Pooled	More prosocial participants are more likely to make prosocial decisions	Yes	Yes	Table S1
3a	Pooled	Discrimination between in- and outgroup members is larger for more prosocial participants as determined by SVO angle	Yes	Yes	Table S1
3b	Meta-Analytic	Discrimination between in- and outgroup members is larger for more prosocial participants as determined by SVO angle	No	Yes, overall effect as expected in 3a, but the directionality of country-level individual effects reverses in some countries	Figure 2, Panel B
4	Pooled	Less effective government (World Bank Governance Indicator) leads to more discrimination in favor of the ingroup	Yes	Yes	Table 1, Figure 3, Panel A
5	Pooled	Decreased importance of religion in everyday life (World Value Survey) predicts increased ingroup favoritism	Yes	No, contrary to the prediction, increased importance of religious institutions was associated with more ingroup-favoring discrimination.	Table 1, Figure 3, Panel B
6	Pooled	Lower historical burden of disease (fewer disability-adjusted life years) are related to increased ingroup favoritism	Yes	Yes	Table 1, Figure 3, Panel C
7	Pooled	Higher COVID-19 deaths are related to increased ingroup favoritism	Yes	Yes	Table 1, Figure 3, Panel D
8	Pooled	In more collectivist cultures (vs. individualist cultures), ingroup favoritism is stronger	Yes	No, contrary to the prediction, increased individualism fostered more ingroup-favoring discrimination	Table 1, Figure 4
9	Pooled	Participants discriminate more when they explicitly prefer learning about matched players' group membership.	No	Yes	Figure 8

Table S10

Overview of hypotheses and results regarding gaze behavior

#	Analysis Type	Hypothesis	Pre- registered	Result as Expected	Reported in
1a-3a	Pooled	Decision effort (decision time, fixation count, information inspected) increases when facing an in- vs. outgroup member	Yes	Yes	Table 2, Figure 5, Panels A-C
1b-3b	Meta-Analytic	Decision effort (decision time, fixation count, information inspected) increases when facing an in- vs. outgroup member	Yes	Yes, overall effects as expected, but the directionality of country-level individual effects reverses in some countries	Figure 6, Panels A-C
4a-6a	Pooled	Larger difference in decision effort (decision time, fixation count, information inspected) facing in- vs. outgroup for more prosocial participants	Yes	No, null effect	Table 2, Figure 5, Panels A-C
4b-6b	Meta-Analytic	Larger difference in decision effort (decision time, fixation count, information inspected) facing in- vs. outgroup for more prosocial participants	No	Partially, overall null effect, but the country-level individual effects are largely significant and of varying directionality	Figure S3, Panels A-C
7a	Pooled	Increasingly prosocial participants allocate more attention to others' payoffs compared to their own	Yes	No, null effect	Table 2, Figure 5, Panel D
7b	Meta-Analytic	Increasingly prosocial participants allocate more attention to others' payoffs compared to their own	Yes	Partially, overall null effect, but the country-level individual effects are largely significant and of varying directionality	Figure 6, Panel D
8a-16a	Pooled	Decision effort (decision time, fixation count, information inspected) linked to government effectiveness, religiosity, historic and COVID-19 health burden (directional predictions see preregistration)	Yes	No, largely null effects	Table 3
8b-16b	Meta-Analytic	Decision effort (decision time, fixation count, information inspected) linked to government effectiveness, religiosity, historic and COVID-19 health burden (directional predictions see preregistration)	No	No, null effects	Page 20
17	Meta-Analytic	Decision effort (decision time, fixation count, information inspected) linked to SVO distribution (proportion of prosocial and altruistic participants; range)	No	No, null effects	Page 20
18	Pooled	Participants visually attend to information revealing others' group membership significantly more often than to the matched players'	No	Yes	Page 21

		random number			
19-22	Pooled	Increasingly prosocial preferences, better liking of and higher identification with their team compared to the outgroup, as well as increasing expectations that others would look up group membership predicted higher odds of looking up group identifying information oneself	Yes (analysis for all, directional hypothesis for identification and liking	Yes	Figure 7, Table S4
23-26	Pooled	Government effectiveness, religiosity, historic and COVID-19 health burden predict visual attention to group identifying information (direction hypotheses see preregistration)	Yes	No, null effects	Table 5
27	Pooled	Explicit preferences to find out others' group membership predict higher visual inattention to group membership information.	Yes (hypothesis only)	Yes	Table S5
